# Complete Genome Analysis of *Pectobacterium brasiliense* BS1113, a Causal Agent of Cigar Tobacco Soft Rot, with Phenotypic Characterization of Virulence and Copper Tolerance

**DOI:** 10.3390/genes17070775

**Published:** 2026-06-30

**Authors:** Xuemei Zhang, Chao Lu, Zhijie Hu, Xiuting Geng, Gang Li, Jian Cai

**Affiliations:** 1 School of Biology and Food Engineering, Fuyang Normal University, Fuyang 236037, China; zhangxm@fynu.edu.cn (X.Z.); 202407024@fynu.edu.cn (C.L.); 201508011@fynu.edu.cn (X.G.); 2 The Second Affiliated Hospital of Tianjin University of Traditional Chinese Medicine, Tianjin 300250, China; hzj2026@sina.com; 3 Joint Research Center for Chinese Herbal Medicine of Anhui ofIHM, Bozhou Vocational and Technical College, Bozhou 236800, China

**Keywords:** *P. brasiliense*, cigar tobacco, soft rot, complete genome, copper tolerance, *T3SS*-deficient

## Abstract

**Background:** *Pectobacterium brasiliense*-mediated soft rot severely threatens the production of diverse cash crops worldwide and brings severe yield reduction risks. A virulent strain BS1113 was separated from diseased cigar tobacco plants collected in Yunnan, yet its virulence regulatory genes and copper resistance-related genetic background have not been fully analyzed so far. This study aims to decipher the genomic features of BS1113 and clarify its pathogenic and copper-tolerant characteristics via whole-genome sequencing, comparative genomics and indoor phenotype verification. **Methods:** Hybrid sequencing strategies combining Illumina short reads and PacBio long reads were adopted to obtain the complete circular genome sequence of strain BS1113. Subsequent comparative genomic analysis and multiple phenotypic identification experiments were conducted to characterize its genetic architecture and physiological traits. **Results:** Genome assembly results showed that the circular chromosome of BS1113 spans 4,916,962 bp with a GC content of 51.96%, which encodes a total of 4369 functional protein-coding genes. Genomic comparison revealed that BS1113 completely lacks the T3SS gene cluster, while it conserves intact T2SS, T6SS and I-F CRISPR-Cas systems; the chromosomal copper resistance operon copRSAB was also detected in this isolate. Pathogenicity tests validated that BS1113 satisfies all criteria of Koch’s postulates on cigar tobacco hosts. In addition, BS1113 displayed prominent tolerance against eight mainstream copper bactericides widely used for tobacco disease management. **Conclusions:** This research generates the first complete high-quality genome of *P. brasiliense* isolated from cigar tobacco hosts. The genomic data explain the infection mechanism of this pathogen independent of intact T3SS, and also reveal the genetic basis supporting its persistent survival under long-term copper fungicide pressure in field cultivation environments.

## 1. Introduction

Soft rot is a destructive bacterial disease caused by *Pectobacterium* and *Dickeya* species, and it severely threatens crop production across the globe [1,2,3,4,5]. After infection, plant tissues develop water-soaked decay and macerated parenchyma, which leads to substantial annual yield losses. Advances in 16S rRNA sequencing and multi-locus biochemical methods have continuously updated the taxonomic status of these soft rot pathogens over recent decades [6]. Originally regarded as a subspecies of *P. carotovorum*, *P. brasiliense* was formally elevated to a separate species in 2019 based on Portier’s systematic genomic and biochemical research [7]. This pathogen can infect a wide variety of hosts, including common solanaceous vegetables and premium cigar tobacco cultivated in southwest China [8,9,10]. Cigar tobacco differs from regular field crops due to its high pectin and lignin content in cell walls. Such structural features form unique defensive barriers and reshape host–pathogen interactions [11,12]. Recent field investigations in Yunnan have found frequent soft rot outbreaks on local cigar tobacco cultivars infected by *P. brasiliense*, which greatly reduced output of the regional tobacco industry [9]. These severe economic losses have driven research into the pathogenic mechanisms, virulence-related genes and agrochemical resistance of this pathogen.

Most publicly released *P. brasiliense* complete genomes are isolated from common vegetable crops, and nearly no genomic data has been reported for cigar tobacco-derived strains, creating a critical research gap in pathogen host adaptation [9]. Genome-wide sequencing technology has become an indispensable tool to dissect virulence determinants and stress-resistance genes of soft rot pathogenic bacteria [13,14]. Copper-containing bactericides are widely adopted for tobacco disease control globally due to their broad-spectrum antibacterial activity [15]. Long-term field application of copper chemicals generates persistent selection pressure on indigenous bacterial populations, driving the emergence of copper-resistant strains and gradually reducing field control efficacy. Copper resistance phenotypes have been extensively characterized in *Xanthomonas* and *Pseudomonas* genera, and recent laboratory tests also verify the intrinsic copper tolerance of most field-collected *Pectobacterium* isolates. However, systematic investigations focusing on *P. brasiliense* strains isolated from cigar tobacco remain scarce [16,17,18]. Further phenotypic identification and genomic excavation of locally resistant isolates can fill this research gap. Multiple secretion systems jointly regulate the pathogenicity of *Pectobacterium* pathogens, among which T2SS, T3SS and T6SS represent three core subtypes with distinct physiological roles during host infection [19]. The T2SS secretes abundant cell wall-degrading enzymes into the extracellular environment to break down plant tissues; the classic T3SS mediates effector protein delivery into host plant cytoplasm, while T6SS mainly mediates interbacterial antagonism and partial infection processes. Pan-genome comparison of multiple strains can uncover sequence polymorphisms in secretion gene clusters, CRISPR-Cas operons and heavy metal resistance loci. Such comparative genomic evidence helps explain pathogen adaptive evolution driven by different host environments and lays a theoretical foundation for the genomic analysis of strain BS1113.

High-quality gap-free genome sequences were obtained from strain BS1113, a pathogen isolated from diseased cigar tobacco leaves in Baoshan, Yunnan. The genome was then compared with seven publicly available *Pectobacterium* reference strains, and multiple in vitro physiological experiments were carried out afterwards. This work intends to characterize virulence gene clusters and the chromosomal copper-resistance operon, reveal the pathogenic pathway independent of T3SS, and expand genomic datasets of cigar tobacco soft rot pathogens native to China.

## 2. Materials and Methods

### 2.1. Bacterial Strains and Genomic DNA Extraction

#### Source of Plant Materials

Here we document how the strain was preserved and where tobacco samples were collected. These settings ensure reliable experimental materials for genomic DNA extraction and subsequent phenotypic analyses. Cigar tobacco (Nicotiana tabacum cv. Yunxue 1) seedlings and leaves used in this study were provided by the cigar tobacco planting base in Lujiang Town, Baoshan City, Yunnan Province, China. The plant materials were cultivated under standard field management conditions for cigar tobacco production. No wild or endangered plant species were involved in this study. All sample collections and experimental procedures complied with local agricultural guidelines and institutional regulations of Fuyang Normal University. Formal identification of the host plant was performed by professional agronomists from the cigar tobacco planting base. No voucher specimens were deposited because the materials were common cultivated cigar tobacco varieties.

Strain BS1113 of *P. brasiliense* was recovered from diseased cigar tobacco leaves sampled in Baoshan, Yunnan. The tobacco cultivar Yunxue 1 is commercially planted in local mountainous areas as a raw material for cigar production. For routine maintenance, the isolate was cultivated using LB medium and stored at −80 °C with 20% sterile glycerol. Genomic DNA was extracted from fresh bacterial pellets using a bacterial genomic DNA extraction kit according to standard protocols. Morphological, physiological, biochemical, and host range assays were performed according to standard procedures for soft rot pathogens (Supplementary File S2: Table S2). High-quality genomic DNA for sequencing can only be obtained from properly maintained microbial strains.

### 2.2. Whole-Genome Sequencing, Hybrid Assembly and Assessment

Hybrid sequencing was performed using Illumina short reads and PacBio long reads. This method successfully generated complete, gapless circular chromosomes. The obtained genomic sequences support further analyses, including gene functional annotation. Whole-genome sequencing was performed using a hybrid strategy combining Illumina NovaSeq (150 bp paired-end short reads) and PacBio RS II long-read sequencing. A 20-kb SMRTbell library was constructed for long-read sequencing. Raw reads were quality-filtered using SMRT Link v10.1. De novo assembly was performed using SMRT Analysis v2.3.0 and Unicycler v4.0.0 with three rounds of polishing to improve accuracy. Genome coverage reached 861.0-fold. The complete genome was visualized as a circular map using CGView v1.1. (Supplementary File S1: Table S1; Supplementary File S5: Table S3; Supplementary File S6: Table S4). Reliable assembled genome sequences are necessary to carry out systematic gene annotation in the next experimental step.

### 2.3. Gene Prediction and Functional Annotation

Genome-wide gene prediction and functional annotation were carried out for strain BS1113. These analyses helped screen candidate genes related to virulence and copper resistance. Protein-coding sequences (CDSs) were predicted using the NCBI Prokaryotic Genome Annotation Pipeline (PGAP). Manual curation of virulence-related genes was performed via BLASTP 2.13.0 searches against UniProtKB/Swiss-Prot, VFDB, and PHI-base. tRNA genes were predicted using tRNAscan-SE v2.0, rRNA genes using RNAmmer v1.2, and sRNA genes using integrated computational pipelines. Functional classification was performed using BLASTP against NR, Pfam, COG, and KEGG databases. Metabolic pathways were mapped using the KAAS server. Signal peptides and transmembrane helices were predicted using SignalP 4.0 and TMHMM 2.0, respectively. Prophage regions were identified using PHASTER, and CRISPR–Cas systems were detected using CRISPRCasFinder v1.0. Antimicrobial resistance genes were annotated using the CARD database. Complete genome annotation lays the foundation for comparative genomic analysis across multiple strains. Corresponding results are provided in the following content.

### 2.4. Comparative Genomic Analysis

Seven *Pectobacterium* genomes were selected as references by considering their taxonomic positions, host backgrounds and reported virulence features. These isolates were recovered from cucumber, potato and Chinese cabbage, covering diverse subspecies of this genus. Pan-genome analysis against these references was performed to explore genetic differences in secretion systems and resistance genes. The comparison focused on BS1113 from cigar tobacco and other *P. brasiliense* strains collected from common vegetables. Plasmid screening using PlasmidFinder 2.1 confirmed that BS1113 carries no extrachromosomal plasmids. Seven complete genomes of *Pectobacterium* strains were downloaded from GenBank for comparison: *P. carotovorum* subsp. *odoriferum* BC S7 (CP009678), *P. brasiliense* SX309 (CP020350), *P. brasiliense* 1692 (CP047495), *P. wasabiae* CFBP 3304 (CP015750), *P. carotovorum* subsp. *carotovorum* PCC21 (CP003776), *P. brasiliense* 21PCA_AGRO2 (CP113504), and *P. carotovorum* subsp. *carotovorum* ICMP 5702 (AODT00000000).

- Average Nucleotide Identity (ANI): calculated using OrthoANIu v1.2
- Digital DNA–DNA hybridization (dDDH): estimated using GGDC 2.1
- Whole-genome alignments and synteny: performed using Mauve v2.3.1 and MUMmer v3.22
- Orthologous gene clustering: conducted using OrthoFinder v2.5.4
- Venn diagrams: constructed in R

CRISPR spacer targets: identified by BLAST 2.13.0 against bacteriophage and plasmid databases. (Supplementary File S7: Table S5; Supplementary File S8: Table S6). Bioinformatic predictions derived from genomic data must be validated via in vitro and in vivo phenotypic experiments.

### 2.5. Pathogenicity Assays and Verification of Koch’s Postulates

Inoculation tests were conducted to validate Koch’s postulates for strain BS1113 and observe soft rot symptoms on detached leaves and whole cigar tobacco seedlings. All pathogenicity assessments were repeated three times biologically. For each replicate, 12 detached leaves and 8 intact seedlings were used. Pathogenicity was tested on cigar tobacco seedlings and detached leaves. Bacterial suspensions (OD_600_ = 0.6) were inoculated onto wounded leaves and stems. Inoculated plants were incubated at 28 °C and high humidity for 3–7 days to observe symptom development. Bacteria were re-isolated from diseased tissues, purified, and identified by colony morphology, Gram staining, and 16S rRNA sequencing to fulfill Koch’s postulates. Pathogenicity tests demonstrated the strong virulence of strain BS1113. Subsequent detection of extracellular hydrolase activities was performed to assess how this strain degrades plant cell walls.

### 2.6. Extracellular Plant Cell Wall-Degrading Enzyme Assays

Quantitative enzyme tests were performed to measure the synthesis of cell-wall-degrading enzymes, which act as major virulence factors in soft rot pathogens. The culture medium for five hydrolase assays was supplemented with 1% corresponding substrates (polypectate, carboxymethyl cellulose), 0.5% peptone and 1.5% agar. Its pH was set to 7.0 before autoclaving [20]. Extracellular hydrolytic enzyme activities were determined on agar plates containing specific substrates:

- Pectate lyase (Pel)
- Polygalacturonase (Peh)
- Cellulase (Cel)
- Protease (Prt)
- β-1,3-glucanase (Glu)Bacterial suspensions (OD_600_ = 0.6; 10 μL per spot) were incubated at 28 °C for 48 h. Hydrolytic halo formation was recorded as evidence of enzyme activity. Besides hydrolases linked to pathogenicity, copper tolerance was also evaluated in subsequent copper resistance tests. This trait serves as an important adaptive characteristic of the strain.

### 2.7. Copper Tolerance Assay

Copper tolerance of BS1113 was assessed via the disk diffusion method in accordance with CLSI guidelines, using eight copper-based fungicides commonly used for local cigar tobacco cultivation. Eight commercial copper-based bactericides and four non-copper antibiotics were tested at recommended field concentrations. Sterile distilled water served as a negative control. Inhibition zone diameters were measured after incubation at 28 °C for 24 h. All treatments included three biological replicates (*n* = 3). Statistical analysis was performed using the Kruskal–Wallis test followed by Dunn’s post hoc test with Bonferroni correction. Table S7 in Supplementary File S9 presents detailed data regarding active ingredients, contents and manufacturers of the copper compounds used in this study. All statistical calculations were completed in GraphPad Prism 9.5. The analyses adopted the Kruskal–Wallis test, alongside Dunn’s post hoc test with Bonferroni correction. Collectively, these phenotypic results corroborate the predicted virulence and copper resistance genes from genomic analysis of BS1113.

## 3. Results

### 3.1. General Genomic Features of P. brasiliense BS1113

A total of 1,361,455,351 clean paired-end Illumina reads (150 bp) and PacBio long reads were obtained for strain BS1113, with a long-read N50 value of 7373 bp. Hybrid genome assembly via Unicycler v4.0.0 yielded one gap-free circular chromosome supported by 861.0-fold sequencing depth (Supplementary File S1: Table S1). The assembled chromosome spans 4,916,962 bp and exhibits a G + C content of 51.96%. Genome structural annotation predicted 4369 protein-coding open reading frames, alongside 77 transfer RNA genes and 22 ribosomal RNA genes (including eight 5S, seven 16S and seven 23S rRNA copies).

**Figure 1:**
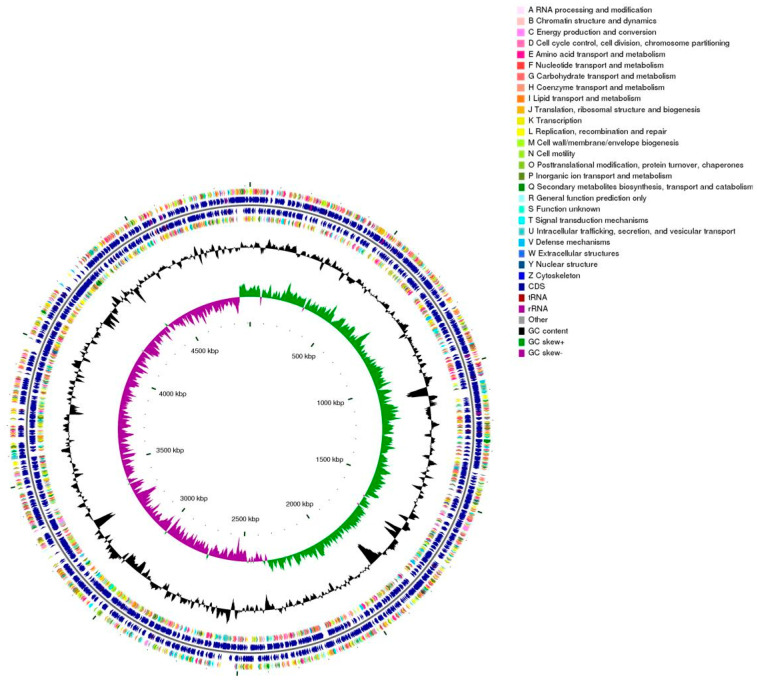
Circular genome map of *P. brasiliense* BS1113 constructed using CGview Server. From outer to inner: CDS on the forward strand (colored by COG category), CDS on the reverse strand, GC content, and GC skew (purple < 0, green > 0).

Genome-wide tandem repeat analysis identified 29 repeat loci, which occupy 0.36% of the total chromosomal sequence (Table 1, Figure 1). Most of these tandem repeats distribute within intergenic intervals adjacent to plant cell wall-degrading enzyme genes, such as polygalacturonase gene0827 and pectate lyase gene1856. One intact prophage region with a length of 18,271 bp and 51.96% G + C content was annotated by PHASTER, harboring 24 coding sequences that mainly encode integrases and uncharacterized hypothetical proteins.

Comparative genomic analysis against publicly available *P. brasiliense* strains revealed that the 4.92 Mb genome of isolate BS1113 is marginally smaller than strain SX309 (4.97 Mb) yet larger than strain 1692 (4.85 Mb) (Table 2). The 51.96% chromosomal G + C ratio of BS1113 falls within the documented range for all sequenced *Pectobacterium* species (50.40–52.18%).

### 3.2. Gene Prediction and Annotation

**Figure 2:**
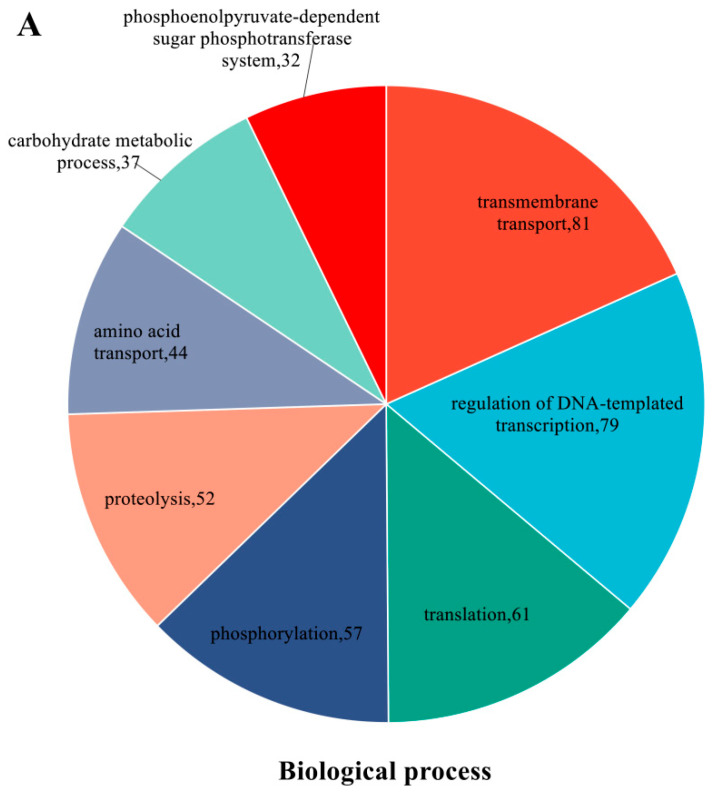

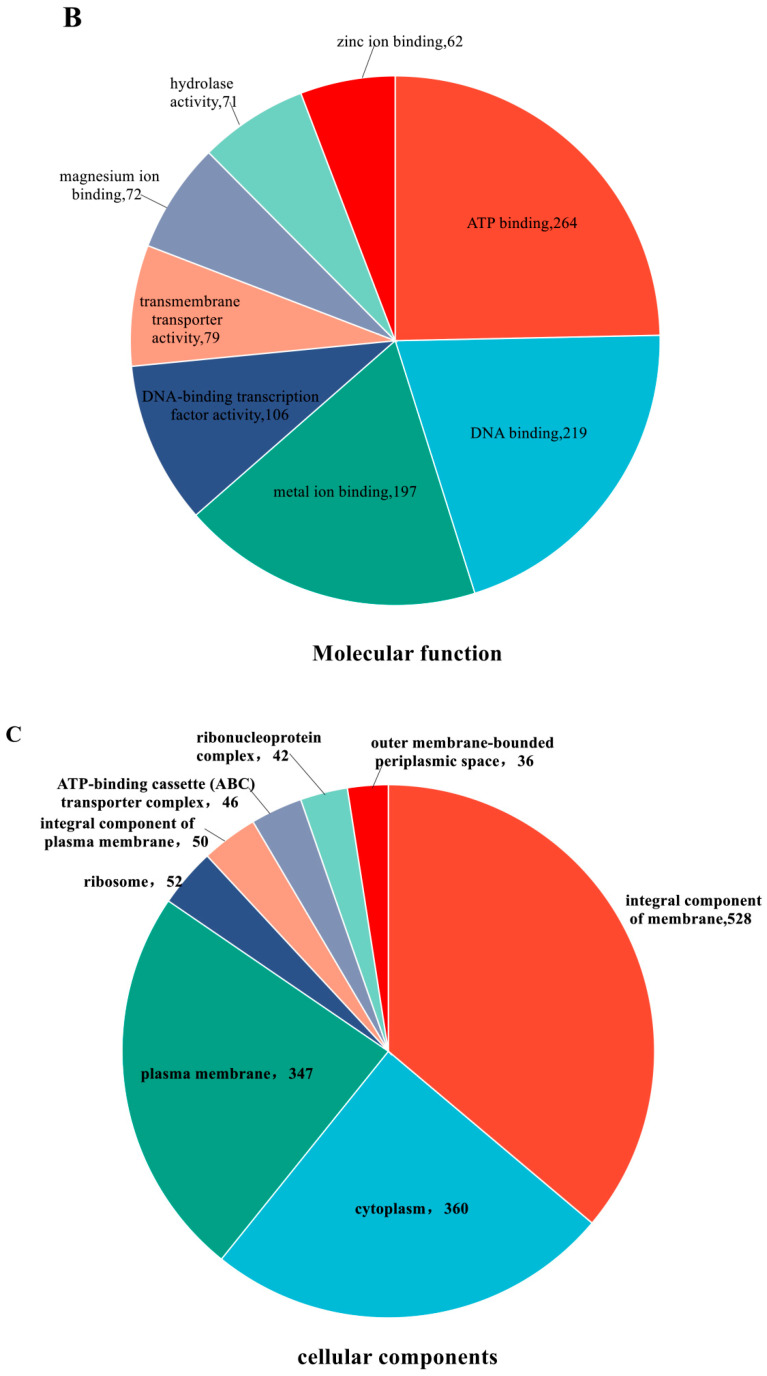
Gene Ontology (GO) classification of protein-coding genes from *P. brasiliense* BS1113. Genes were assigned to three categories: biological process (**A**), molecular function (**B**), and cellular component (**C**).

A total of 4369 CDSs and 149 sRNAs were predicted. Gene Ontology (GO) analysis assigned 2298 genes to three core categories (Figure 2). Within the molecular function category, ‘ATP binding’ was the most frequent term (*n* = 264, 6.04%), followed by ‘DNA binding’ (*n* = 219, 5.01%), ‘Metal ion binding’ (*n* = 197, 4.51%), and ‘DNA-binding transcription factor activity’ (*n* = 106, 2.43%). In the cellular component category, ‘Integral component of membrane’ was the largest group (*n* = 528, 12.09%), followed by ‘Cytoplasm’ (*n* = 360, 8.24%) and ‘Plasma membrane’ (*n* = 347, 7.94%). For biological processes, ‘transmembrane transport’ (*n* = 81, 1.85%) was the most frequent, followed by ‘regulation of DNA-templated transcription’ (*n* = 79, 1.81%) and ‘translation’ (*n* = 61, 1.40%).

**Figure 3:**
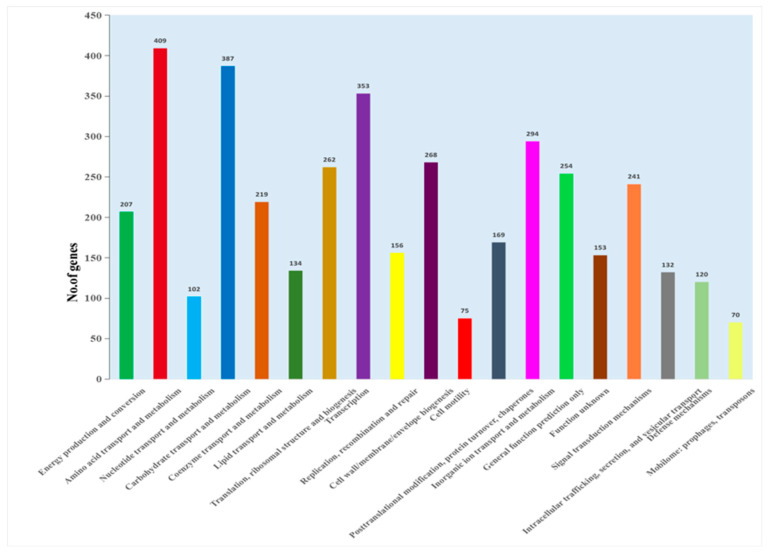
Cluster of Orthologous Groups (COG) functional annotation of CDS from *P. brasiliense* BS1113. A total of 4520 CDS were classified into 19 functional categories.

BLASTP searches against the COG database classified 3690 genes into 24 functional categories (Figure 3). The four largest categories were ‘Transcription’ (*n* = 353, 9.56%), ‘Inorganic ion transport and metabolism’ (*n* = 294, 7.96%), ‘Cell wall/membrane/envelope biogenesis’ (*n* = 268, 7.26%), and ‘Energy production and conversion’ (*n* = 207, 5.61%). ‘Cell motility’ (*n* = 75), ‘Transport and catabolism’ (*n* = 66), and ‘Defense mechanisms’ (*n* = 120) were the least represented categories. ‘Amino acid transport and metabolism’ (*n* = 409, 11.08%) and ‘Carbohydrate transport and metabolism’ (*n* = 387, 10.49%) were well represented.

**Figure 4:**
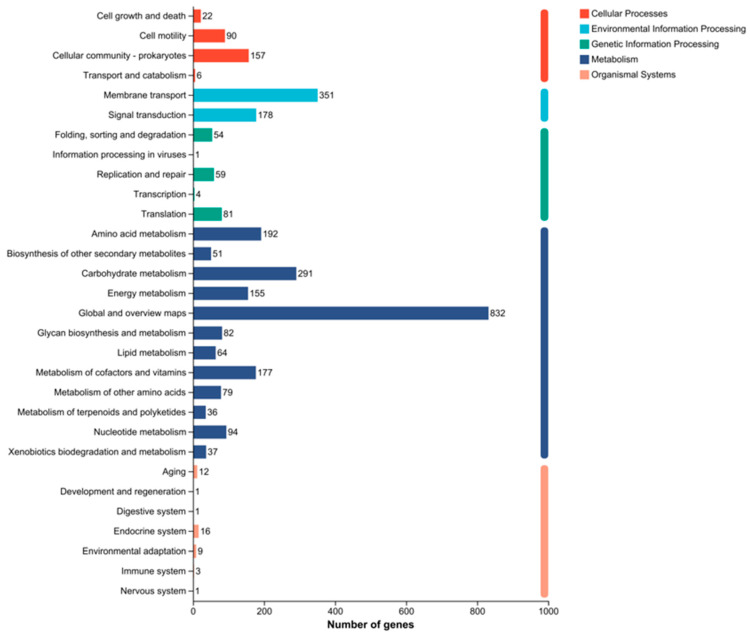
KEGG pathway assignment of protein-coding genes from *P. brasiliense* BS1113. Genes were grouped into five major functional categories.

Of the 4369 annotated genes, 2897 (*n* = 2897, 66.3%) were assigned to KEGG pathways (Figure 4); only bacterial pathways were retained, while all non-prokaryotic classification entries were excluded. ‘Metabolism’ accounted for the largest number of genes (*n* = 1993, 45.6%), followed by ‘Global and overview maps’ (*n* = 832, 19.0%), ‘Carbohydrate metabolism’ (*n* = 291, 6.7%), and ‘Amino acid metabolism’ (*n* = 192, 4.4%). Within ‘Environmental information processing’ (*n* = 529, 12.1%), ‘Membrane transport’ (*n* = 351, 8.0%) and ‘Signal transduction’ (*n* = 178, 4.1%) were the dominant subcategories. Under ‘Cellular processes’ (*n* = 275, 6.3%), ‘Cellular community—prokaryotes’ (*n* = 157, 3.6%) and ‘Cell motility’ (*n* = 90, 2.1%) were the most frequent.

Combined analyses identified 634 putative virulence-associated proteins, including PCWDEs (e.g., polygalacturonase gene0827, pectate lyase gene1856) and T2SS/T6SS components (e.g., VasD, *Gsp*E). CARD analysis identified seven CDSs with homology to known antibiotic resistance determinants, including genes associated with fluoroquinolones, carbapenems, diaminopyrimidines, phenicols, and cephalosporins (Supplementary File S6). CRISPR-Cas detection (CRISPRCasFinder) revealed six CRISPR repeat regions in BS1113: the longest (2013 bp) contained 34 spacers, while the shortest (92 bp) contained 2 spacers. Twelve spacers showed sequence homology to phages reported from tobacco-associated environments (e.g., *Erwinia* phage phiEa2809) (Supplementary File S8).

### 3.3. Comparative Genomics of P. brasiliense BS1113 with Other Pectobacterium Strains

Seven complete genomes of *Pectobacterium* strains were included for comparison (Table 2). All strains carry a single chromosome without plasmids.

**Figure 5:**
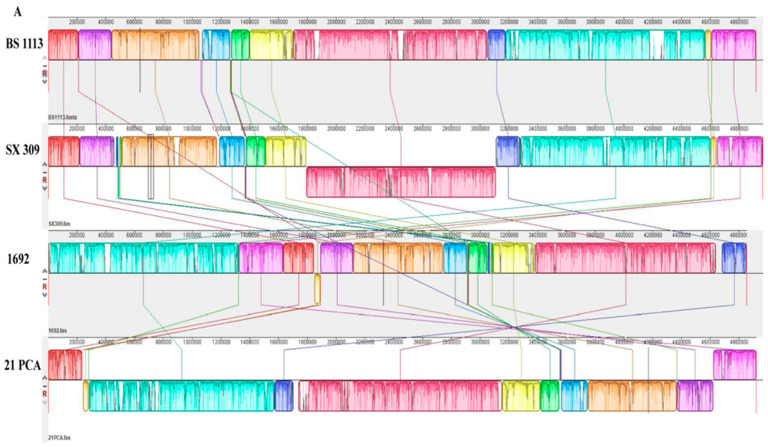

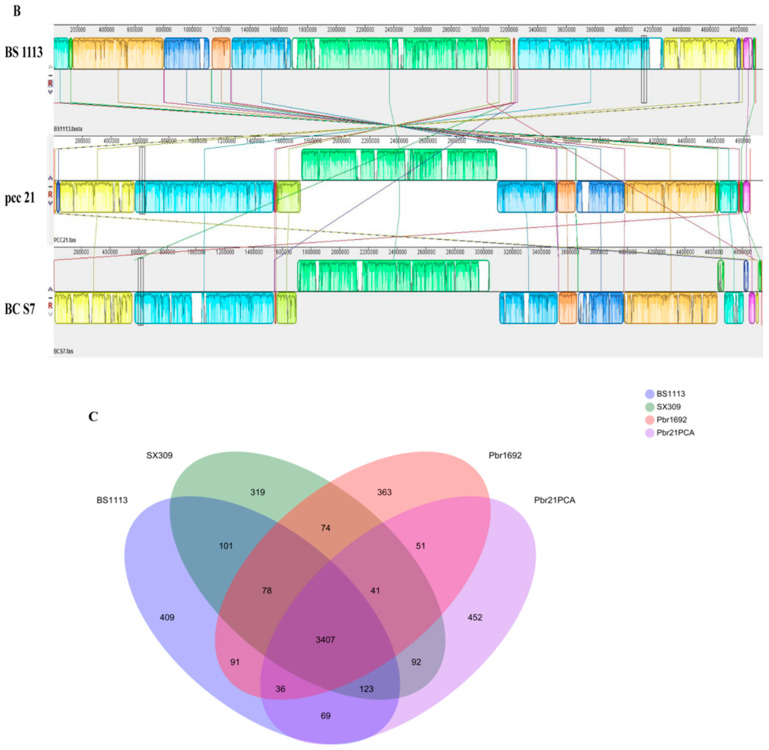

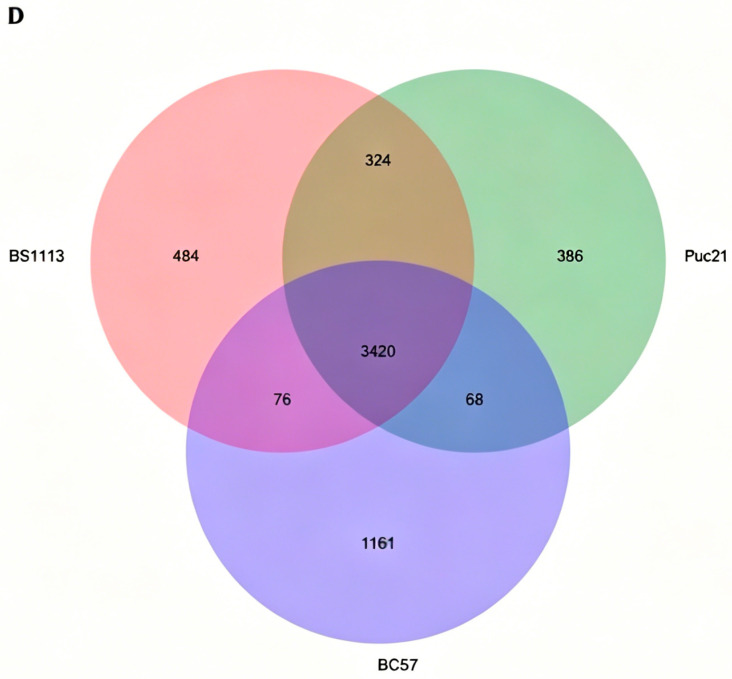
Whole-genome comparison of *P. brasiliense* BS1113 with related *Pectobacterium* strains. (**A**) Mauve alignment of BS1113 with SX309, 1692, and 21PCA. (**B**) Mauve alignment at the subspecies level. (**C**,**D**) Venn diagrams showing orthologous gene clusters.

Whole-genome alignments (Mauve v2.3.1) showed that BS1113 shares higher sequence similarity with SX309 than with strains 1692 or 21PCA, consistent with ANI (96.8%) and dDDH (72.3%). At the subspecies level, BS1113 showed greater synteny with *P. carotovorum* subsp. *carotovorum* PCC21 than with *P. carotovorum* subsp. *odoriferum* BC S7 (Figure 5A,B). Pairwise comparison between BS1113 and PCC21 identified no large insertions/deletions (>5 kb), but three large local collinear block (LCB) inversions were detected: one of −87 kb encompassing a PCWDE-enriched region (gene0827, gene1856) and two flanking T6SS core genes (vasD, *clpV*).

**Figure 6:**
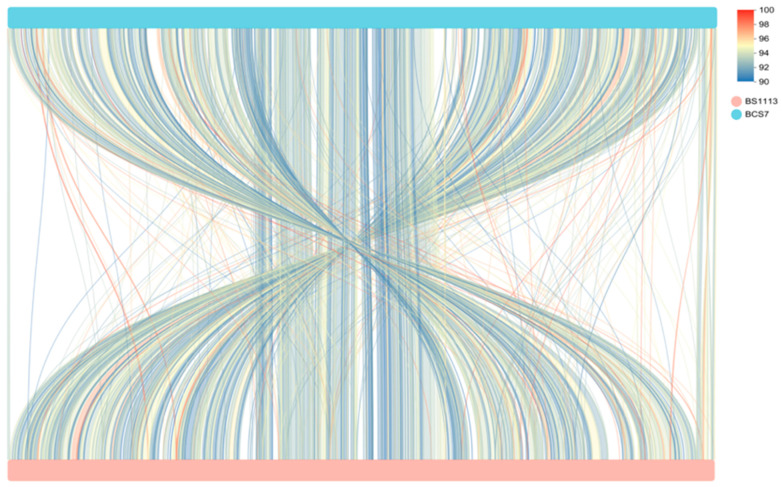
Whole-genome synteny comparison between *P. brasiliense* BS1113 and *P. carotovorum* subsp. *odoriferum* BCS7. The vertical tracks at the top and bottom represent the full-length chromosomes of BS1113 (red) and BCS7 (blue), respectively. Colored connecting lines indicate homologous collinear regions, and the color gradient bar on the right corresponds to pairwise sequence identity (ranging from 90% to 100%).

Orthologous clustering (OrthoFinder v2.5.4, default parameters) showed that the core genome of BS1113, SX309, 1692 and 21PCA comprises 3407 orthologous genes. BS1113 has 409 unique gene families (Figure 5C). At the subspecies level (BS1113, BC S7, PCC21), 3420 orthologous genes are shared; BS1113 shares 78 genes with BC S7 and 324 genes with PCC21, and has 494 unique gene families (Figure 5D). The unique gene families in BS1113 are enriched in GO terms ‘lipoprotein localization to membrane’ (GO:0044873) and ‘DNA modification’ (GO:0006304). Whole-genome synteny plots (MUMmer) revealed partial synteny between BS1113 and BC S7, with multiple inversions, translocations, and deletions (Figure 6).

### 3.4. Pathogenicity Assays and Fulfillment of Koch’s Postulates

To confirm that *P. brasiliense* BS1113 is the causal agent of soft rot on cigar tobacco, Koch’s postulates were performed using two inoculation methods as described in Section 2.

Leaf inoculation. Healthy cigar tobacco leaves (cv. ‘Yunxue 1’) were inoculated with a bacterial suspension (10^8^ CFU/mL, OD_600_ = 0.6) by the pin-prick method. Within 24 h post-inoculation, water-soaked lesions appeared on inoculated leaves, which expanded into necrotic, macerated tissue by 48 h. Control leaves inoculated with sterile LB broth remained asymptomatic (Supplementary File S4: Figure S2C,D).

Stem inoculation by capillary method. The stem base of tobacco seedlings was inoculated with the same bacterial suspension using a capillary tube. At 48 h post-inoculation, inoculated plants showed wilting, stem rot, and internal discoloration of the stem pith, whereas control plants remained healthy (Supplementary File S4: Figure S2A,B,E,F).

Re-isolation. Bacteria re-isolated from symptomatic leaf and stem tissues formed colonies identical to the original isolate on NA plates, were Gram-negative rods, and were confirmed as *P. brasiliense* by 16S rRNA gene sequencing (99.8% identity). No bacteria were recovered from control plants. These results fulfill Koch’s postulates for strain BS1113 as a causal agent of soft rot on cigar tobacco.

### 3.5. Extracellular Plant Cell Wall-Degrading Enzyme (PCWDE) Activities

The production of major extracellular hydrolytic enzymes by BS1113 was assessed on substrate-specific agar plates (see Section 2). After 48 h of incubation at 28 °C, clear zones (halos) indicating enzyme activity were observed around the inoculation spots for pectinase (on pectin-containing plate, stained with CTAB), cellulase (on CMC plate, stained with Congo red), and β-1,3-glucanase (on laminarin plate). No detectable protease activity was observed on skim milk agar under the tested conditions. The largest and most distinct clearing zones were consistently produced on pectinase plates, indicating strong pectinolytic activity. Representative images of the enzyme activity assays are shown in Supplementary File S11: Figure S4. Three independent experiments yielded consistent results.

### 3.6. Plant Cell Wall-Degrading Enzyme Genes

The genome of BS1113 encodes multiple PCWDEs, including four polygalacturonases (gene0827, gene1319, gene3148, gene3251), two pectin acetylesterases (gene1166, gene2259), two pectate lyases (gene1856, gene4417), and eleven β-glucosidases (Table 3). One gene encoding β-xylosidase (gene2616) and one encoding oligogalacturonate lyase (gene1974) were also identified.

### 3.7. Secretion Systems

**Figure 7:**
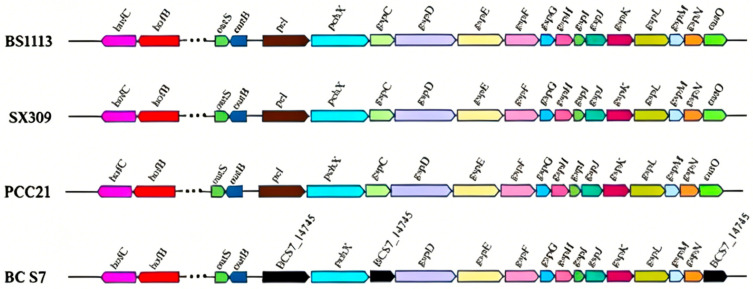
Genetic organization of the type II secretion system (T2SS) gene cluster in *Pectobacterium* strains. Arrows indicate transcriptional direction.

The genome of *P. brasiliense* BS1113 encodes multiple secretion systems. A complete type II secretion system (T2SS) gene cluster was identified, spanning 17,669 bp and comprising 15 open reading frames: gspC, gspD, gspE, gspF, gspG, gspH, gspI, gspJ, gspK, gspL, gspM, gspN, outO, outS, and outB (Figure 7). The predicted Gsp proteins share 92–100% amino acid identity with orthologs in *P. brasiliense* SX309 and *P. carotovorum* subsp. *carotovorum* PCC21, and 85–98% identity with those in *P. carotovorum* subsp. *odoriferum* BC S7 (Supplementary File S12: Table S8). A 120-bp insertion located upstream of gspE contains a predicted PhoP-PhoQ binding motif (MEME Suite). Genes of the Sec-SRP system are present in BS1113, with the exception that secA and secE are absent in BC S7 (Supplementary File S12: Table S8).

No genes encoding a type III secretion system (*T3SS*) were detected. BLASTn searches against the *T3SS* reference database (T3DB, E < 1 × 10^−5^) and manual inspection of the *hrp* locus region confirmed the complete absence of *hrp* and *hrc* gene clusters in BS1113.

**Figure 8:**
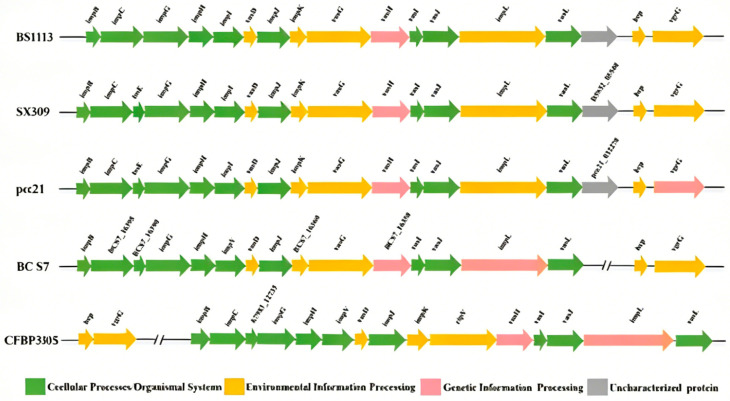
Structural organization of the type VI secretion system (T6SS) gene cluster in *Pectobacterium* strains. Colored arrows represent conserved core components.

A type VI secretion system (T6SS) gene cluster comprising 33 genes was identified. This cluster contains 15 core structural genes (vasD, impL, *clpV*, impB, impC, tssE, impG, impH, impI, impJ, vasH, vasI, vasJ, vasL, tssI) and five vgrG/thirteen hcp effector-encoding genes (Figure 8). Comparative analysis showed that the copy numbers of vgrG (5 in BS1113 vs. 3 in SX309) and hcp (13 vs. 10) differ among strains (Supplementary File S13: Table S9).

### 3.8. Copper Tolerance Determinants and Phenotypic Validation

Screening the BS1113 genome against CARD (e-value < 1 × 10^−5^) identified a putative copper tolerance operon (locus tags ACU36R_11035 to ACU36R_11050), comprising copS (histidine kinase), copR (response regulator), copA (P1B-type ATPase), and copD (copper-binding protein). All four genes share the same transcriptional orientation.

**Figure 9:**
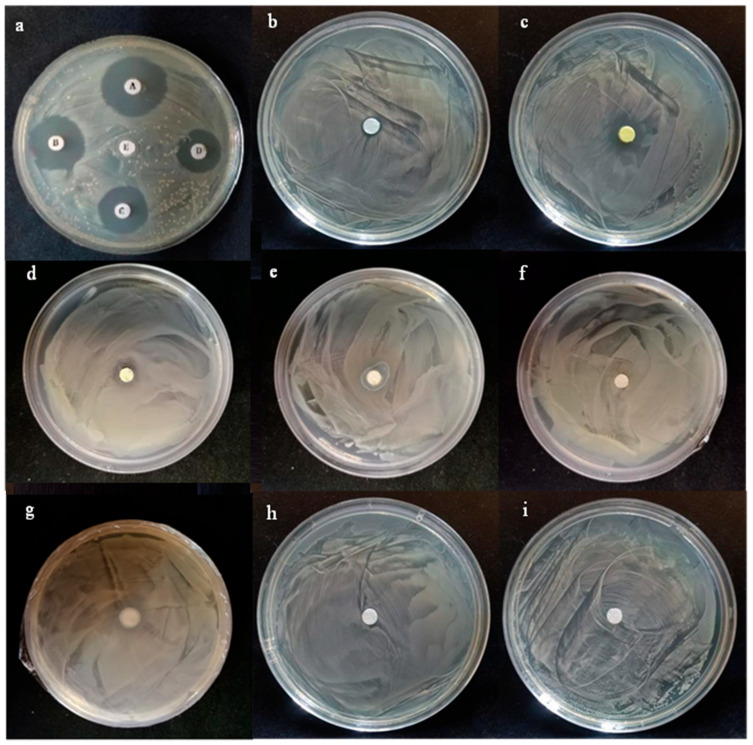
Tolerance of *P. brasiliense* BS1113 to 12 bactericides (8 copper-based and 4 non-copper). Inhibition zone diameters were measured after 24 h incubation at 28 °C. Data are means ± SD (*n* = 3). Asterisks indicate significant differences (*p* < 0.01) determined by the Kruskal–Wallis test followed by Dunn’s post hoc test with Bonferroni adjustment. (**a**) Four non-copper bactericides and blank control: A = kasugamycin, B = ethylicin, C = zhongshengmycin, D = sodium dichloroisocyanurate, E = blank control (CK); (**b**) Copper abietate; (**c**) Kasugamycin·copper quinolate; (**d**) Copper succinate, glutarate and adipate; (**e**) Copper succinate, glutarate and adipate; (**f**) Thiodiazole copper; (**g**) Mixed lipid·copper sulfate; (**h**) Zinc thiazole; (**i**) Nonglian Kangyousu.

The copper tolerance phenotype of BS1113 was assessed by disc diffusion assay using eight commercial copper-based bactericides and four non-copper antibiotics at the manufacturers’ recommended field concentrations (Supplementary File S4: Table S4). BS1113 showed no measurable inhibition zone for any of the eight copper-based formulations (0 mm for all). In contrast, all four non-copper antibiotics produced clear inhibition zones: kasugamycin (16.3 ± 1.2 mm), ethylicin (21.5 ± 0.8 mm), zhongshengmycin (12.7 ± 0.9 mm), and sodium dichloroisocyanurate (18.4 ± 1.1 mm) (mean ± SD, *n* = 3). No inhibition zones were observed for any of the eight copper-based bactericides tested, indicating high copper tolerance in vitro. The overall comparison (Kruskal–Wallis test, followed by Dunn’s post hoc test with Bonferroni adjustment) showed a significant difference among the 12 treatments (*p* < 0.01). Post hoc comparisons confirmed that each non-copper antibiotic produced significantly larger inhibition zones than any copper-based product (adjusted *p* < 0.05 for all pairwise comparisons) (Figure 9).

### 3.9. Two-Component Systems (TCSs)

The BS1113 genome encodes 19 two-component systems (Supplementary File S13: Table S9). Based on the domain architecture of the histidine kinase and response regulator, these were classified into five subfamilies: OmpR (nine pairs), NarL (five pairs), CitB (two pairs), NtrC (two pairs), and chemotaxis (one pair). The PhoP-PhoQ system (locus tags ACU36R_09665–09670) is present and shares >95% amino acid identity with orthologs in other *Pectobacterium* strains. The GacA/GacS system was not detected in BS1113. Additional TCSs identified include regulators involved in phosphate starvation (PhoR/B), envelope stress (CpxA/R, BaeS/R), anaerobic respiration (ArcB/A), motility (CheA/Y), capsular synthesis (RcsC/D), potassium limitation (KdpD/E), osmotic stress (EnvZ/OmpR), nitrogen assimilation (GlnL/G), as well as systems of unknown function (RstB/A, BasS/R) (Supplementary File S14: Table S10).

### 3.10. CRISPR-Cas Systems

**Figure 10:**
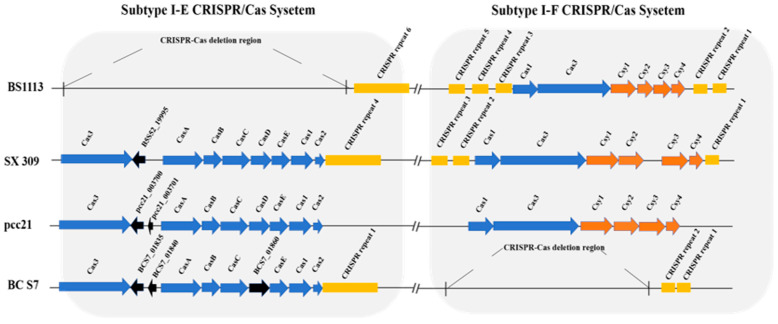
Organization of CRISPR-Cas loci in selected *Pectobacterium* strains. Blue: subtype I-F; orange: subtype I-E; yellow: CRISPR repeat regions.

CRISPR-Cas systems were identified in the genomes of four *Pectobacterium* strains (Supplementary File S14: Table S10 and Figure 10). BS1113 carries a single subtype I-F system containing cas1, cas3, csy1, csy2, csy3, and csy4. *P. brasiliense* SX309 and *P. carotovorum* subsp. *carotovorum* PCC21 each contain two subtypes (I-F and I-E). *P. carotovorum* subsp. *odoriferum* BC S7 carries a single subtype I-E system. The number of CRISPR repeats varies: PCC21 lacks detectable repeats, whereas BS1113, SX309 and BC S7 each contain three or more repeats of different lengths. BLAST analysis of spacer sequences revealed homology to phages infecting *Pectobacterium, Erwinia*, and *Ralstonia*, as well as to various plasmids (Supplementary File S15: Table S11).

## 4. Discussion

This study reports the complete genome and phenotypic characteristics of *P. brasiliense* strain BS1113, isolated from diseased cigar tobacco. Consistent with previous reports on *Pectobacterium* species, BS1113 lacks the canonical type III secretion system (T3SS) but remains pathogenic on cigar tobacco, confirming that T3SS is not essential for virulence in this pathosystem. Phylogenetic and comparative genomic analyses (ANI > 96%, dDDH > 70%) place BS1113 within the *P. brasiliense* clade, consistent with previous taxonomic studies [7,21,22,23]. The genome is 4.92 Mb in size with a G + C content of 51.96%, and contains 4369 protein-coding sequences. Phenotypic assays confirmed that BS1113 causes soft rot symptoms on cigar tobacco, fulfilling Koch’s postulates, and produces extracellular pectinase, cellulase, and β-1,3-glucanase activities.

### 4.1. Absence of the Type III Secretion System

A notable genomic feature of BS1113 is the complete absence of the *hrp* and *hrc* gene clusters encoding the T3SS. This finding distinguishes BS1113 from several other sequenced *P. brasiliense* strains, including SX309, which contain a complete T3SS [14]. However, the presence of a T3SS is not universally required for pathogenicity in *Pectobacterium*. Kim et al. (2009) [24] reported that most T3SS-deficient *Pectobacterium* strains exhibited virulence levels comparable to T3SS-containing strains in plant assays. Multiple recent genome analyses conducted in 2024 further indicated that sporadic loss of T3SS fragments existed in field-isolated *P. brasiliense* strains from cash crops. Such gene loss was mainly caused by long-term host niche selection pressure [23]. Therefore, the absence of T3SS in BS1113 does not necessarily imply a fundamentally different pathogenic strategy; rather, it represents a naturally occurring genomic variation within the species [24]. Recent multiple genomic reports have identified several naturally T3SS-deficient *P. brasiliense* strains isolated from different cash crops. These isolates vary in infection aggressiveness and preferred host ranges despite retaining intact PCWDE-dependent pathogenic pathways [23,24].

The factors that might compensate for the lack of T3SS in BS1113 remain unknown. One possibility is that a wound-dependent field infection mode and robust PCWDE secretion via intact T2SS may fully compensate for T3 effector-mediated infection. This trait corresponds to the unique high-pectin cell wall structure of cigar tobacco and supports pathogen proliferation after tissue maceration [25]. However, direct experimental comparisons between BS1113 and a T3SS-containing strain (e.g., SX309) on the same host would be required to test this hypothesis.

### 4.2. Plant Cell Wall-Degrading Enzymes and the Type II Secretion System

The genome of BS1113 encodes a full set of PCWDEs, including four polygalacturonases, two pectate lyases, and eleven β-glucosidases (Table 3). These enzymes are properly folded in the cytoplasm and then transported across bacterial cells via the T2SS channel proteins. Each structural subunit of T2SS contributes to the assembly of secretion pores for targeted extracellular secretion [26]. As core virulence determinants of soft rot pathogens, T2SS-mediated secretion of PCWDEs drives host cell wall decomposition and tissue maceration [19,27,28]. Compared with ordinary vegetables, cigar tobacco has high pectin content in its parenchymal cells. The diverse pectin-degrading enzymes encoded by BS1113 allow the strain to efficiently degrade host polysaccharides [9]. Phenotypic assays confirmed that BS1113 produces detectable pectinase, cellulase, and β-1,3-glucanase activities on agar plates (Supplementary File S11: Figure S4). These observations are consistent with the established role of PCWDEs and T2SS in soft rot pathogenesis [1,12,29]. Quantitative comparisons of PCWDE activity or expression between BS1113 and other strains were not performed in this study and remain a subject for future investigation.

### 4.3. Type VI Secretion System and CRISPR-Cas

The T6SS gene cluster in BS1113 contains 33 genes, including 15 core structural genes and five vgrG/thirteen hcp effector-encoding genes. The copy numbers of vgrG (5) and hcp (13) differ from those in SX309 (3 vgrG, 10 hcp) (Supplementary File S12: Table S8). The T6SS mainly performs antibacterial activity by delivering toxic effectors into neighboring bacterial cells to occupy field ecological niches. Variations in effector gene copy numbers represent an evolutionary adaptation to the complex bacterial community associated with tobacco plants [30,31]. BS1113 carries a single subtype I-F CRISPR-Cas system, whereas SX309 and PCC21 each contain two subtypes (I-F and I-E). The I-F-type CRISPR-Cas complex is widely conserved across *Pectobacterium* species. This molecular system can recognize invading phages and degrade their foreign nucleic acids [9]. The CRISPR spacers of strain BS1113 show high similarity to genetic sequences from phages inhabiting local tobacco fields. Long-term phage pressure in natural fields enables the strain to obtain new spacer sequences and strengthen its immune defense during field adaptation. This observation is consistent with the acquisition of spacers from local phage populations, but does not by itself indicate a niche-specific adaptive advantage.

### 4.4. Two-Component Systems

BS1113 lacks the GacA/GacS two-component system, which is present in many *Pectobacterium* strains and has been reported to regulate T3SS and other virulence factors [32,33]. The absence of GacA/GacS, together with the absence of T3SS, is a notable genomic difference between BS1113 and SX309. When the canonical Gac regulatory cascade is absent, the conserved PhoP-PhoQ and CpxA/R two-component systems take over the regulation of various PCWDE genes [26]. The PhoP-PhoQ system monitors changes in the periplasm, while CpxA/R perceives cell envelope stress. Their combined activities sustain stable transcription of hydrolase genes and support the strain’s pathogenic traits independent of Gac control. All 19 characterized two-component systems are described in detail in Supplementary File S10: Figure S3. Whether and how BS1113 regulates PCWDE expression in the absence of GacA/GacS is unknown; related gene knockout verification will be arranged in subsequent research plans.

### 4.5. Copper Tolerance

The BS1113 genome contains a copS-copR-copA-copD gene cluster, which has been described in other *Pectobacterium* genomes [18,34]. On a molecular basis, CopS serves as a membrane-bound histidine kinase. This protein responds to extracellular copper and relays phosphorylation signals to cytoplasmic CopR, which in turn initiates transcription of the downstream *copA* and *copD* genes. *CopA* removes surplus copper ions from the bacterial cell, while *CopD* sequesters free intracellular copper to mitigate toxic effects [35]. Disc diffusion assays showed that no inhibition zones were observed for any of the eight copper-based bactericides tested, whereas four non-copper antibiotics produced clear inhibition zones (Figure 9), matching the functional annotation of this chromosomal resistance operon. PlasmidFinder analysis confirmed that BS1113 carries no extrachromosomal genetic elements. Its copper resistance trait is encoded on the chromosome, rather than mobile genetic material. Frequent application of copper-based bactericides in local cigar fields imposes long-term selective pressure, which keeps the complete *cop cluster* stably present in the bacterial genome [34]. but this interpretation remains speculative without temporal or population-level data.

### 4.6. Limitations of the Study

Several limitations should be acknowledged. First, although Koch’s postulates were fulfilled for BS1113 on cigar tobacco, comparative pathogenicity assays with *T3SS*-containing strains (e.g., SX309) on the same host were not performed. Therefore, whether the absence of *T3SS* affects virulence level, host range, or infection dynamics remains untested. Second, the inferred functions of T6SS, CRISPR-Cas, and specific TCSs are based solely on genomic annotation and literature comparison; no knockout or complementation experiments have been conducted. Third, the enzymatic activities of PCWDEs were assessed qualitatively on plate assays; quantitative measurements (e.g., specific activity, kinetic parameters) were not performed. Fourth, the observation of a unique gene repertoire and syntenic differences between BS1113 and other strains (e.g., LCB inversions) does not demonstrate functional adaptation; experimental validation is required. Fifth, the evolutionary history of *T3SS* loss in BS1113 cannot be determined from a single genome; comparative genomics of additional cigar tobacco isolates would be needed to assess whether this trait is common or sporadic.

## 5. Conclusions

Overall, this work describes the complete genome of *P. brasiliense* strain BS1113, a causal agent of cigar tobacco soft rot. The genome lacks the T3SS gene cluster but carries intact T2SS, T6SS, PCWDEs, CRISPR-Cas, and copper tolerance loci. Phenotypic assays confirm strong pathogenicity and high copper tolerance. This genomic resource provides a foundation for investigating virulence evolution, host adaptation, and fungicide resistance mechanisms in *P. brasiliense* associated with cigar tobacco.

This study provides several novel findings regarding the virulence and chemical resistance of the cigar tobacco-derived pathogen *P. brasiliense* strain BS1113. First, we obtained the complete closed circular genome of BS1113, which represents the first high-quality genomic dataset of *P. brasiliense* linked to cigar tobacco soft rot in China. Second, genomic and phenotypic evidence revealed that this isolate naturally lacks the intact T3SS gene cluster yet retains strong pathogenic capacity, which is mainly driven by plant cell wall degrading enzymes and functional T2SS machinery. Third, the chromosomal copper-resistance operon *copRSAB* was identified as the key genetic element enabling BS1113 to withstand eight widely used copper bactericides applied in tobacco field management. Collectively, these genomic resources expand our understanding of the infection and stress adaptation mechanisms of soft rot pathogens infecting specialty tobacco crops.

## Figures and Tables

**Table 1:** Genomic features of *P. brasiliense* BS1113.

Attribute	Value
Genome Size	4,916,962
Shape of DNA	Circular
No. of coding genes	4369
Maximum sequence length	214,063
Average sequence length	6831.44
N50 length	7373
%GC	51.96
Gene Density(%)	86.67
No. of tandem repeats	29
In Genome (%)	0.36
Number of sRNAs:	149
No. of transfer RNAs(tRNAs)	77
No. of ribosomal RNAs (rRNAs)	22

**Table 2:** Genomic features of *P. brasiliense* BS1113 and other *Pectobacterium* spp.

Features	BS1113	BC S7	1692	SX309	*Pcc* 21	21PCA	CFBP3304
Size (bp)	4,916,962	4,933,575	4,851,982	4,966,299	4,842,771	4,919,671	5,043,228
G + C content (%)	51.96	51.80	52.15	52.18	52.18	51.67	50.55
Replicons	One chromosome	One chromosome	One chromosome	One chromosome	One chromosome	One chromosome	One chromosome
Total genes	4468	4868	4310	4455	4340	4506	4579
Predicted no. of CDS	4369	4868	4205	4351	4263	4407	4472
Ribosomal RNA	22	22	22	22	22	22	22
Transfer RNA	77	77	77	76	76	77	77
GenBank sequence	CM128641.1	CP009678.1	CP047495.1	CP020350.1	CP003776.1	CP113504.1	CP015750.1

**Table 3:** Cell wall-degrading enzyme gene statistics.

Class Definition	Protein Code	Gene ID	
polygalacturonase	(EC 3.2.1.15)	gene0827 gene1319 gene3148 gene3251	
pectin acetyl esterase (PAGE)	(EC 3.1.1.-)	gene1166 gene2259	
pectate lyase	(EC 4.2.2.2)	gene1856 gene4417	
exopolygalacturonate lyase	(EC 4.2.2.9)	gene1856 gene4417	
beta-glucosidase	(EC 3.2.1.21)	gene0012 gene0035 gene2923 gene3513 gene3723 gene0735	gene2264 gene2568 gene2616 gene2796 gene2894
oligogalacturonate lyase	(EC 4.2.2.6)	gene1974	
beta-xylosidase	(EC 3.2.1.37)	gene2616	

## Data Availability

The complete genome sequence of *P. brasiliense* strain BS1113 was deposited in GenBank under the accession number CM128641.1. All other data generated or analyzed during this study are included in this published article and its Supplementary Materials.

## Supplementary Materials

The following supporting information can be downloaded at: https://www.mdpi.com/article/10.3390/genes17070775/s1. Table S1 Project information and sequencing statistics for *Pectobacterium brasiliense* BS1113. Table S2 Classification and general features of *Pectobacterium brasiliense* strain BS1113 according to the MIGS recommendations. Table S3 Project information for *Pectobacterium brasiliense* strain BS1113. Table S4 Genome statistics. Table S5 The locus tag information of 16S rRNA genes and five housekeeping genes used for phylogenetic analysis in this study. Table S6 Percentage of average nucleotide identities (ANI)a and in silico DNA-DNA hybridization (DDH)b among the selected *Pectobacterium* genomes. Table S7 Tested fungicides. Table S7 Identification of homologs of type II and Sec-SRP secretion system genes in *P. brasiliense* BS1113 and other *Pectobacterium* spp. Table S8 Genetic elements of T6SS-encoding gene clusters in pathogenic *Pectobacterium* spp. were summarized and the presence of the key T6SS structure genes is indicated for the analysed genomes. Table S9 Homolog of two-component system encoding genes in *P. brasiliense* BS1113 and other *Pectobacterium* spp. Table S10 Homologs of Clustered regularly interspaced short palindromic repeats (CRISPR)-CRISPR-associated protein (Cas) in *P. brasiliense* BS1113 and other *Pectobacterium* spp.
