# Supplementary material for: Complete Genome Analysis of Pectobacterium brasiliense BS1113, a Causal Agent of Cigar Tobacco Soft Rot, with Phenotypic Characterization of Virulence and Copper Tolerance
Source: Genes (Basel). 2026 Jun 30;17(7):775. doi: 10.3390/genes17070775 (PMC13408941; doi:10.3390/genes17070775)
Supplement: Supplementary file 1 [file genes-17-00775-s001.zip › Additional file 7.pdf]

Table S5 The locus tag information of 16S rRNA genes and five housekeeping genes used for phylogenetic analysis in this study.

Strain (aName)	16S rRNA 16S ribosomal RNA	*gap*A Glyceraldehyde-3-phosphate dehydrogenase	*gyr*A DNA gyrase subunit A	*atp*D ATP synthase chain beta	*rpo*A DNA-directed RNA polymerase, subunit alpha	*rho* Transcription termination factor Rho
BS1113	ACU36R_RS01730	ACU36R_RS13815	ACU36R_RS14445	ACU36R_RS04835	ACU36R_RS13790	ACU36R_RS01180
SX309	B5S52_02585	B5S52_11815	B5S52_15995	B5S52_22070	B5S52_02510	B5S52_01150
BC1	NC16_16860	NC16_10010	NC16_05460	NC16_21345	NC16_19015	NC16_20030
BZA12	CTV95_13730	CTV95_04540	CTV95_08950	CTV95_15110	CTV95_17635	CTV95_16480
PC1	PC1_R0067	PC1_1965	PC1_1965	PC1_4257	PC1_3797	PC1_4011
PCC21	PCC21_rRNA000200	PCC21_020260	PCC21_042390	PCC21_042390	PCC21_037850	PCC21_039900
BC S7	BCS7_00865	BCS7_10145	BCS7_05730	BCS7_21390	BCS7_18930	BCS7_20060
Q106	KT343626	BV921_13355	BV921_09485	BV921_16725	BV921_22205	BV921_18195
ICMP 5702	G032_18350	G032_12315	G032_05770	G032_20745	G032_18375	G032_19445
DSM 30168	NR_041971	SAMN05444147_101280	SAMN05444147_103367	SAMN05444147_107231	SAMN05444147_11815	SAMN05444147_108192
KKH3	NR_125539	KKH3_19450	KKH3_09580	KKH3_42220	KKH3_37100	KKH3_39650
ICMP 19972	BSK71_08965	BSK71_13255	BSK71_03400	BSK71_18250	BSK71_20780	BSK71_16715
SCC1	SCC1_0194	SCC1_2178	SCC1_3166	SCC1_4402	SCC1_0439	SCC1_0231
SCC3193	W5S_3637	W5S_2202	W5S_3209	W5S_4734	W5S_4136	W5S_4366
CFBP 3304	A7983_06320	A7983_18675	A7983_18675	A7983_07720	A7983_05005	A7983_06160

SCRI1043	16S_rRNA-1	ECA2344	ECA1201	ECA4512	ECA4006	ECA4211
JG10-08	EV46_19855	EV46_11280	EV46_0604	EV46_22460	EV46_19930	EV46_21005
21A	GZ59_43070	GZ59_22140	GZ59_12300	GZ59_46110	GZ59_40620	GZ59_42750
WPP163	Pecwa_R0095	Pecwa_2261	Pecwa_3211	Pecwa_454	Pecwa_3975	Pecwa_3975
RNS08.42.1A	A8F97_17225	A8F97_07325	A8F97_02595	A8F97_18700	A8F97_21605	A8F97_20465
NIBIO1006	BJJ97_00415	BJJ97_15115	BJJ97_10920	BJJ97_04970	BJJ97_02650	BJJ97_03670
EC1	W909_01010	W909_09290	W909_05450	W909_20185	W909_1805	W909_19005
Ech586	Dd586_R0001	Dd586_2162	Dd586_1081	Dd586_4159	Dd586_3711	Dd586_3900
IPO2222	A4U42_03735	A4U42_19205	A4U42_14785	A4U42_08840	A4U42_06715	A4U42_07640
ND14b	LH89_10945	LH89_01940	LH89_06440	LH89_12580	LH89_14780	LH89_13845
Ech1591	Dd1591_R0100	Dd1591_2215	Dd1591_3019	Dd1591_4194	Dd1591_0348	Dd1591_0157
3937	Dda3937_04220	Dda3937_03335	Dda3937_01774	Dda3937_00145	Dda3937_01515	Dda3937_00267
Ech703	Dd703_R0005	Dd703_1973	Dd703_1039	Dd703_3993	Dd703_0430	Dd703_0204
CFBP1430	EAMY_r01	EAMY_1976	EAMY_2345	EAMY_3702	EAMY_3361	EAMY_0170
ATCC 49946	EAM_r001	EAM_1931	EAM_2264	EAM_3474	EAM_3173	EAM_0163
Ep1/96	EpC_r22	EpC_16320	EpC_12850	EpC_36910	EpC_33500	EpC_01840
Eb661	EbC_08400_tr07	EbC_24210	EbC_30400	EbC_45870	EbC_41050	EbC_01950
Et1/99	ETA_r010	ETA_15590	ETA_12260	ETA_34750	ETA_31380	ETA_01960
Ejp617	EJP617_r003	EJP617_30660	EJP617_34080	EJP617_11650	EJP617_08420	EJP617_13820
DSM 12163	EPYR_r001	EPYR_01755	EPYR_01369	EPYR_03975	EPYR_03610	EPYR_00193
EM595	EM595_r001	EM595_2009	EM595_2486	EM595_3461	EM595_0393	EM595_3337

Strain name reference: BS1113: *Pectobacterium brasiliense* BS1113 (this study); SX309: *Pectobacterium brasiliense* SX309; BC1: *Pectobacterium brasiliense* BC1;

BZA12: *Pectobacterium brasiliense* BZA12; PC1: *Pectobacterium aroidearum* PC1; PCC21: *Pectobacterium carotovorum* subsp. *carotovorum* PCC21; BC S7: *Pectobacterium carotovorum* subsp. *odoriferum* BC S7; Q106: *Pectobacterium carotovorum* subsp. *odoriferum* Q106; ICMP 5702: *Pectobacterium carotovorum* subsp. *carotovorum* ICMP 5702; DSM 30168: *Pectobacterium carotovorum* subsp. *carotovorum* DSM 30168; KKH3: *Pectobacterium carotovorum* subsp. *actinidiae* KKH3; ICMP 19972: *Pectobacterium carotovorum* subsp. *actinidiae* ICMP 19972; SCC1: *Candidatus Pectobacterium* maceratum SCC1; SCC3193: *Pectobacterium parmentieri* SCC3193; CFBP 3304: *Pectobacterium wasabiae* CFBP 3304; SCRI1043: *Pectobacterium atrosepticum* SCRI1043; JG10-08: *Pectobacterium atrosepticum* JG10-08; 21A: *Pectobacterium atrosepticum* 21A; WPP163: *Pectobacterium parmentieri* WPP163; RNS08.42.1A: *Pectobacterium parmentieri* RNS08.42.1A; NIBIO1006: *Pectobacterium polaris* NIBIO1006; EC1: *Dickeya* zeae EC1; Ech586: *Dickeya* zeae Ech586; IPO2222: *Dickeya* solani IPO2222; ND14b: *Dickeya* solani ND14b; Ech1591: *Dickeya chrysanthem*i Ech1591; 3937: *Dickeya dadantii* 3937; Ech703: *Dickeya paradisiaca* Ech703; CFBP1430: *Erwinia amylovora* CFBP1430; ATCC 49946: *Erwinia amylovora* ATCC 49946; Ep1/96: *Erwinia pyrifoliae* Ep1/96; Eb661: *Erwinia billingiae* Eb661; Et1/99: *Erwinia tasmaniensis* Et1/99; Ejp617: *Erwinia* sp. Ejp617; DSM 12163: *Erwinia pyrifoliae* DSM 12163; EM595: *Erwinia gerundensis* EM595.
