# Supplementary material for: Complete Genome Analysis of Pectobacterium brasiliense BS1113, a Causal Agent of Cigar Tobacco Soft Rot, with Phenotypic Characterization of Virulence and Copper Tolerance
Source: Genes (Basel). 2026 Jun 30;17(7):775. doi: 10.3390/genes17070775 (PMC13408941; doi:10.3390/genes17070775)
Supplement: Supplementary file 1 [file genes-17-00775-s001.zip › Additional file 9.pdf]

Table S7 Tested fungicides

Fungicide	Trade name	Manufacturer
Kasugamycin 20% WP	Hairun Wuyan	Sichuan Hairun Crop Science and Technology Co., Ltd.
Ethylicin 80% EC	Shinaijing	Kaifeng Dadi Agrochemical Biotechnology Co., Ltd.
Zhongshengmycin 3% WP	Mi'er	Sichuan Jinzhu Ecological Agriculture Technology Co., Ltd.
Sodium dichloro isocyanurate20%WP	Dika	Weihai Hanfu Biochemical Pharmaceutical Co., Ltd.
Resin acid copper salt23% EW	Manfu Manor	Shandong Heyi Biotechnology Co., Ltd.
Zinc thiazole30% SC	Qianyun	Zhejiang Xinnong Chemical Co., Ltd.
Chunlei * quinoline copper33% SC	Lüte (Lvte)	Shandong Yilan Technology Co., Ltd.
Cupric thiazide20% SC	Jiatian	Zhejiang Longwan Chemical Co., Ltd.
Mixed fat *copper sulfate24% EC	Dongwang Duxiao	Hubei Nongben Chemical Co., Ltd.
Copper(succinate+glutarate+adipate)30% SC	Saoxi	Hebei Zhongbao Lvnong Crop Technology Co., Ltd.
Copper hydroxide77% WG	Minglan	Jiangsu Mingde Lida Crop Technology Co., Ltd.
Thiosencopper 20%SC	Shibeijun	Zhejiang Dongfeng Chemical Co., Ltd.
