# Supplementary material for: Complete Genome Analysis of Pectobacterium brasiliense BS1113, a Causal Agent of Cigar Tobacco Soft Rot, with Phenotypic Characterization of Virulence and Copper Tolerance
Source: Genes (Basel). 2026 Jun 30;17(7):775. doi: 10.3390/genes17070775 (PMC13408941; doi:10.3390/genes17070775)
Supplement: Supplementary file 1 [file genes-17-00775-s001.zip › Additional file 12.pdf]

Table S7 Identification of homologs of type II and Sec-SRP secretion system genes in *P.brasiliense* BS1113 and other *Pectobacterium* spp.

Genes in BS1113*	Accesion no. in BS1113	SX309		PCC21		BC S7	
		Accesion no.	Homology (%)	Accesion no.	Homology (%)	Accesion no.	Homology (%)
**Type II secretion system**							
*hof*C	WP_015731233.1	ARA75021.1	98	AFR04974.1	98	AIU89729.1	95
gspC	WP_015841050.1	ARA75662.1	99	AFR04337.1	97	[a]NA	[a]NA
gspD	WP_010300146.1	ARA75663.1	99	AFR04336.1	98	AIU89204.1	96
gspE	WP_005969987.1	ARA75664.1	99	AFR04335.1	98	AIU89203.1	96
gspF	WP_005969986.1	ARA75665.1	99	AFR04334.1	98	AIU89202.1	95
gspG	WP_014916099.1	ARA75666.1	100	AFR04333.1	99	AIU90534.1	97
gspH	WP_015841045.1	ARA75667.1	98	AFR04332.1	96	AIU89201.1	95
gspI	WP_015841044.1	ARA75668.1	99	AFR04331.1	98	AIU89200.1	97
gspJ	WP_011094627.1	ARA75669.1	98	AFR04330.1	97	AIU90533.1	95
gspK	WP_010302966.1	ARA75670.1	99	AFR04329.1	98	AIU89199.1	96
gspL	WP_010681642.1	ARA75671.1	99	AFR04328.1	98	AIU89198.1	94
gspM	WP_014916093.1	ARA75672.1	99	AFR04327.1	98	AIU89197.1	92
gspN	WP_015841039.1	ARA75673.1	97	AFR04326.1	96	AIU89196.1	93
gspO	WP_010302946.1	ARA75674.1	97	AFR04325.1	96	[a]NA	[a]NA

gspS	WP_014916107.1	ARA75658.1	100	AFR04341.1	99	AIU89207.1	95
gspB	WP_014916106.1	ARA75659.1	99	AFR04340.1	97	AIU89206.1	85
**Sec-SRP secretion system**							
*sec*A	WP_446729987.1	ARA75015.1	99	AFR04981.1	99	[a]NA	[a]NA
*sec*B	WP_014913761.1	ARA78220.1	100	AFR01564.1	99	AIU86872.1	98
*sec*D	WP_071822569.1	ARA77381.1	100	AFR02457.1	100	AIU87657.1	98
*sec*E	WP_010285488.1	ARA78176.1	100	AFR01608.1	99	AIU86912.1	99
*sec*F	WP_043901963.1	ARA77380.1	100	AFR02458.1	100	[a]NA	[a]NA
*sec*G	WP_014914113.1	ARA77799.1	100	AFR01998.1	100	AIU87260.1	98
*sec*M	WP_446729986.1	ARA75014.1	100	AFR04982.1	100	AIU89735.1	94
*sec*Y	WP_010286083.1	ARA74820.1	100	AFR05193.1	100	AIU89910.1	100
*yaj*C	WP_010282033.1	ARA77382.1	100	AFR02456.1	100	AIU87656.1	100
*yid*C	WP_039281389.1	ARA78443.1	99	AFR05610.1	98	AIU90380.1	98
*fts*Y	WP_446729874.1	ARA74460.1	96	AFR05514.1	95	AIU90215.1	86

[a]NA = not available
