# Supplementary material for: Complete Genome Analysis of Pectobacterium brasiliense BS1113, a Causal Agent of Cigar Tobacco Soft Rot, with Phenotypic Characterization of Virulence and Copper Tolerance
Source: Genes (Basel). 2026 Jun 30;17(7):775. doi: 10.3390/genes17070775 (PMC13408941; doi:10.3390/genes17070775)
Supplement: Supplementary file 1 [file genes-17-00775-s001.zip › Additional file 13.pdf]

Table S8 Genetic elements of T6SS-encoding gene clusters in pathogenic *Pectobacterium* spp. were summarized and the presence of the key T6SS structure genes is indicated for the analysed genomes.

Functional context	Key genes Name	Description	Accesion no. in BS1113	Accesion no. in SX309	Accesion no. in PCC21	Accesion no. in BC S7
**Secreted substrate**	*vgr*G	Type VI secretion system secreted protein VgrG	WP_039517996.1	B5S52_00350<br>B5S52_05200<br>B5S52_05550<br>B5S52_09335<br>B5S52_10725	PCC21_032250<br>PCC21_033020<br>PCC21_041400	BCS7_14575<br>BCS7_16260<br>BCS7_16635<br>BCS7_19685<br>BCS7_20930

	hcp	Type VI secretion system effector, Hcp1 family	WP_014916572.1<br>WP_014916345.1<br>WP_039466971.1<br>WP_039496946.1<br>WP_010285305.1<br>WP_012822145.1	B5S52_00355<br>B5S52_04220<br>B5S52_05420<br>B5S52_05450<br>B5S52_05545<br>B5S52_09330<br>B5S52_13980<br>B5S52_14140<br>B5S52_16680<br>B5S52_18295<br>B5S52_18460<br>B5S52_19450<br>B5S52_21035	PCC21_032260<br>PCC21_041390	BCS7_04320<br>BCS7_14585<br>BCS7_16265<br>BCS7_16310<br>BCS7_20925
**Outer membrane protein**	*vas*D	Type VI secretion system protein VasD (Putative lipoprotein)	WP_010282515.1	B5S52_05495	PCC21_032360	BCS7_14495<br>BCS7_16370
**Inner membrane protein**	*imp*L	Type VI secretion system protein ImpL (IcmF-related protein)	WP_225584378.1	B5S52_05530	PCC21_032290	BCS7_04365<br>BCS7_14545<br>BCS7_16335
	*imp*K	Type VI secretion system protein ImpK/ DotU	WP_014916353.1	B5S52_05505	PCC21_032340	BCS7_14595

**ATPase**	*clp*V	Type VI secretion ATPase, ClpV1 family	WP_320699795.1	B5S52_05510	PCC21_032330	BCS7_04325<br>BCS7_14580<br>BCS7_16355
**Regulatory protein/Other structure protein**	*imp*B	Type VI secretion system protein ImpB	WP_010282535.1	B5S52_05465	PCC21_032420	BCS7_04295<br>BCS7_14610<br>BCS7_16400
	*imp*C	Type VI secretion system protein ImpC	WP_446730115.1	B5S52_05470	PCC21_032410	BCS7_04300<br>BCS7_14605
	*tss*E	Type VI secretion system protein	WP_005972060.1	B5S52_05475	PCC21_032400	BCS7_16390
	*imp*G	Type VI secretion system protein ImpG	WP_014916355.1<br>WP_014916356.1	B5S52_05480	PCC21_032390	BCS7_14505<br>BCS7_16385
	*imp*H	Type VI secretion system protein ImpH	WP_320699795.1	B5S52_05485	PCC21_032380	BCS7_14500<br>BCS7_16380
	*imp*I	Type VI secretion system protein ImpI	WP_205569382.1	B5S52_05490	PCC21_032370	BCS7_16375
	*imp*J	Type VI secretion system protein ImpJ	WP_010282515.1	B5S52_05500	PCC21_032350	BCS7_04305<br>BCS7_14600<br>BCS7_16365

	*vas*H	Sigma-54 dependent transcriptional regulator	ARA75370.1	B5S52_05515	PCC21_032320	[a]NA
	*vas*I	Type VI secretion system protein VasI	WP_014916350.1	B5S52_05520	PCC21_032310	BCS7_13230<br>BCS7_16345
	*vas*J	Type VI secretion system protein VasJ	WP_446730116.1	B5S52_05525	PCC21_032300	BCS7_14540<br>BCS7_16340
	*vas*L	Type VI secretion system protein VasL	WP_205605458.1	B5S52_05535	PCC21_032280	BCS7_14475<br>BCS7_16330
	*tss*I	Type VI secretion protein TssI	WP_446729914.1	[a]NA	[a]NA	[a]NA

[a]NA = not available.
