# Supplementary material for: Complete Genome Analysis of Pectobacterium brasiliense BS1113, a Causal Agent of Cigar Tobacco Soft Rot, with Phenotypic Characterization of Virulence and Copper Tolerance
Source: Genes (Basel). 2026 Jun 30;17(7):775. doi: 10.3390/genes17070775 (PMC13408941; doi:10.3390/genes17070775)
Supplement: Supplementary file 1 [file genes-17-00775-s001.zip › Additional file 14.pdf]

Table S9 Homolog of two-component system encoding genes in *P. brasiliense* BS1113 and other *Pectobacterium* spp.

Genes in BS1113 *	Product sequence or definition in BS1113	Accesion no. or position in BS1113	SX309		PCC21		BC S7	
			Accesion no.	Homology (%)	Accesion no.	Homology (%)	Accesion no.	Homology (%)
*pho*R	Two-component system, OmpR family, phosphate regulon sensor histidine kinase PhoR	WP_039275418.1	ARA77392.1	100	AFR02446.1	98	AIU87646.1	98
*pho*B	Two-component system, OmpR family, phosphate regulon response regulator PhoB	WP_005975981.1	ARA77393.1	100	AFR02445.1	100	AIU87645.1	100
*pho*Q	Two-component system, OmpR family, sensor histidine kinase PhoQ	WP_014915245.1	ARA76629.1	100	AFR03329.1	99	AIU88365.1	96
*pho*P	Two-component system, OmpR family, response regulator PhoP	WP_039469188.1	ARA76628.1	100	AFR03330.1	99	AIU88366.1	96
*env*Z	Two-component system, OmpR family, osmolarity sensor histidine kinase EnvZ	WP_039277203.1	ARA74670.1	99	AFR05292.1	98	AIU90004.1	97
*omp*R	Two-component system, OmpR family, phosphate regulon response regulator OmpR	WP_005969424.1	ARA74669.1	100	AFR05293.1	99	AIU90005.1	98
*rst*B	Two-component system, OmpR family, sensor histidine kinase RstB	WP_446729215.1	ARA76244.1	99	AFR03753.1	99	[a]NA	[a]NA
*rst*A	Two-component system, OmpR family, response regulator RstA	WP_010276861.1	ARA76245.1	100	AFR03752.1	99	AIU88691.1	98

*cpx*A	Two-component system, OmpR family, sensor histidine kinase CpxA	WP_010281575.1	ARA74496.1	100		AFR05479.1	100	AIU90172.1	98
*cpx*R	Two-component system, OmpR family, response regulator CpxR	WP_010281574.1	ARA74497.1	100		AFR05478.1	100	AIU90173.1	98
*bae*S	Two-component system, OmpR family, sensor histidine kinase BaeS	WP_446730176.1	ARA75590.1	99		AFR04412.1	98	AIU89271.1	94
*bae*R	Two-component system, OmpR family, response regulator BaeR	WP_039498485.1	ARA75589.1	98		AFR04413.1	97	AIU89272.1	96
*qse*C	Two-component system, OmpR family, sensor histidine kinase QseC	WP_446729836.1	ARA78390.1	99		AFR05658.1	98	AIU90343.1	93
*qse*B	Two-component system, OmpR family, response regulator QseB	WP_349896008.1	ARA78389.1	99		AFR05659.1	98	AIU90344.1	96
*kdp*D	Two-component system, OmpR family, sensor histidine kinase KdpD	WP_446729419.1	ARA77211.1	99		AFR02649.1	98	AIU87806.1	96
*kdp*E	Two-component system, OmpR family, KDP operon response regulator KdpE	WP_010309408.1	ARA77212.1	100		AFR02648.1	100	[a]NA	[a]NA
*rcs*F	Rcs stress response system protein RcsF	WP_010681579.1	ARA75273.1	100		AFR04748.1	100	AIU89550.1	98
*rcs*C	Two-component system, NarL family, capsular synthesis sensor histidine kinase RcsC	WP_205590978.1	ARA77299.1	99		AFR02540.1	98	AIU87722.1	95
*rcs*D	Two-component system, NarL family, sensor histidine kinase RcsD	WP_039502924.1	ARA77297.1	99		AFR02542.1	98	AIU87724.1	94
*arc*B	Two-component system, OmpR family, aerobic respiration control sensor histidine kinase ArcB	WP_039522240.1	ARA78084.1	99		AFR01718.1	99	AIU87015.1	97

*arc*A	Two-component system, OmpR family, aerobic respiration control protein ArcA	WP_010300511.1	ARA74929.1	100	AFR05070.1	99	AIU89810.1	98
*dcu*S	Two-component system, CitB family, sensor histidine kinase DcuS	ARA74406.1	ARA74406.1	99	AFR05563.1	99	AIU90265.1	99
*dcu*R	Two-component system, CitB family, response regulator DcuR	WP_010281107.1	ARA74407.1	100	AFR05562.1	100	AIU90264.1	99
*nar*X	Two-component system, NarL family, nitrate/nitrite sensor histidine kinase NarX	WP_174435727.1	ARA76260.1	99	AFR03737.1	98	AIU90500.1	96
*nar*L	Two-component system, NarL family, nitrate/nitrite response regulator NarL	WP_010276892.1	ARA76259.1	100	AFR03738.1	100	AIU88684.1	98
*nar*Q	Two-component system, NarL family, nitrate/nitrite sensor histidine kinase NarQ	WP_446730545.1	ARA76060.1	99	AFR03921.1	98	AIU90500.1	29
*nar*P	Two-component system, NarL family, nitrate/nitrite response regulator NarP	WP_446729259.1	ARA76061.1	99	AFR03920.1	98	AIU88772.1	98
*fus*K	Two-component system, NarL family, sensor histidine kinase FusK	AFR02803.1	ARA77056.1	99	AFR02803.1	98	AIU87910.1	97
*fus*R	Two-component system, NarL family, response regulator FusR	AFR02804.1	ARA78553.1	100	AFR02804.1	100	AIU87911.1	100
*gln*L	Two-component system, NtrC family, nitrogen regulation sensor histidine kinase GlnL	WP_010298136.1	ARA78608.1	99	AFR01418.1	98	AIU90362.1	100
*gln*G	Two-component system, NtrC family, nitrogen regulation response regulator	WP_014913637.1	ARA78370.1	100	AFR01417.1	100	AIU90361.1	99

	GlnG							
*glr*K	Two-component system, NtrC family, sensor histidine kinase GlrK	WP_002208627.1	ARA75511.1	99	AFR04492.1	98	AIU89333.1	98
*glr*R	Two-component system, NtrC family, response regulator GlrR	WP_010280116.1	ARA75513.1	100	AFR04490.1	99	AIU89331.1	98
*che*A	Two-component system, chemotaxis family, sensor kinase CheA	WP_446729309.1	ARA75866.1	100	AFR04120.1	98	AIU88947.1	96
*che*Y	Two-component system, chemotaxis family, chemotaxis protein CheY	WP_010279519.1	ARA75871.1	100	AFR04115.1	99	AIU88942.1	99

[a]NA = not available.
