# Supplementary material for: Complete Genome Analysis of Pectobacterium brasiliense BS1113, a Causal Agent of Cigar Tobacco Soft Rot, with Phenotypic Characterization of Virulence and Copper Tolerance
Source: Genes (Basel). 2026 Jun 30;17(7):775. doi: 10.3390/genes17070775 (PMC13408941; doi:10.3390/genes17070775)
Supplement: Supplementary file 1 [file genes-17-00775-s001.zip › Additional file 15.pdf]

Table S10 Homologs of Clustered regularly interspaced short palindromic repeats (CRISPR)-CRISPR-associated protein (Cas) in *P. brasiliense* BS1113 and other *Pectobacterium* spp.

Genes in BS1113*	Product sequence or definition in BS1113	Accesion no. in BS1113	SX309		PCC21		BC S7	
			Accesion no.	Homology (%)	Accesion no.	Homology (%)	Accesion no.	Homology (%)
*csy*4	Type I-F CRISPR-associated endoribonuclease Csy4	WP_446730013.1	ARA75082.1	99	AFR04907.1	98	[a]NA	[a]NA
*csy*3	Type I-F CRISPR-associated protein Csy3	WP_446730014.1	ARA75083.1	100	AFR04906.1	99	[a]NA	[a]NA
*csy*2	Type I-F CRISPR-associated protein Csy2	WP_349842501.1	ARA75130.1	99	AFR04905.1	98	[a]NA	[a]NA
*csy*1	Type I-F CRISPR-associated protein Csy1	WP_446730015.1	ARA75131.1	97	AFR04904.1	96	[a]NA	[a]NA
*cas*3	Type I-F CRISPR-associated helicase Cas3	WP_446730016.1	ARA75132.1	99	AFR04903.1	98	[a]NA	[a]NA
*cas*1	Subtype I-F CRISPR-associated endonuclease Cas1	WP_446730017.1	ARA75133.1	99	AFR04902.1	98	[a]NA	[a]NA

[a]NA = not available.
