# Supplementary material for: Complete Genome Analysis of Pectobacterium brasiliense BS1113, a Causal Agent of Cigar Tobacco Soft Rot, with Phenotypic Characterization of Virulence and Copper Tolerance
Source: Genes (Basel). 2026 Jun 30;17(7):775. doi: 10.3390/genes17070775 (PMC13408941; doi:10.3390/genes17070775)
Supplement: Supplementary file 1 [file genes-17-00775-s001.zip › Additional files.docx.pdf]

Additional file 3:

Figure S1. General morphological characteristics of *Pectobacterium brasiliense* strain BS1113.(A) Transmission electron micrograph (TEM) of a negatively stained BS1113 cell, showing typical rod-shaped morphology (scale bar = 500 nm).(B) Transmission electron micrograph (TEM) of negatively stained BS1113 cells, displaying abundant peritrichous flagella (scale bar = 5 μm).(C) Scanning electron micrograph (SEM) of BS1113 cells harvested at the exponential growth phase (scale bar = 10.0 μm).(D) Scanning electron micrograph (SEM) of BS1113 cells harvested at the stationary growth phase (scale bar = 3.00 μm).

Additional file 4:

Figure S2. Pathogenicity symptoms caused by *Pectobacterium brasiliense* strain BS1113 on cigar tobacco (*Nicotiana tabacum* L.).(A) Healthy control plant (mock-inoculated with sterile LB broth, stem base).(B) Inoculated plant showing wilting and stem rot at 48 h post-inoculation (capillary method, stem base).(C) Healthy control leaf.(D) Inoculated leaf showing water-soaked lesion at 24 h (pin-prick method).(E) Systemic infection and stem rot on an inoculated plant (capillary method, 3 d).(F) Longitudinal sections of tobacco stems: left – inoculated (showing internal soft rot and discoloration); right – healthy control.

Additional file 8:

Table S5. Percentage of average nucleotide identities (ANI)[a] and in silico DNA-DNA hybridization (DDH)[b] among the selected *Pectobacterium* genomes

Additional file 9:

Figure S3. Dot-plot analysis of linear genomic organization between *Pectobacterium brasiliense* strain BS1113 and six other previously fully sequenced Pectobacterium genomes. The X-axis represents the BS1113 genome. The Y-axis represents (A) *P. brasiliense* SX309 (CP020350.1), (B) *P. brasiliense* Pbr1682 (CP047495.1), (C) *P. wasabiae* CFBP 3304 (CP015750.1) and (D) *P. odoriferum* BC S7 (CP009678.1) , respectively. Red indicates aligned sequences in the forward direction; blue indicates aligned sequences in the reverse direction.

Additional file 10:

Figure S4. Extracellular enzyme activities of *Pectobacterium brasiliense* strain BS1113.Plate assays showing the production of extracellular enzymes: cellulase (Cel), protease (Prt), pectinase (Pel/Peh, combined as Pec), and β-1,3-glucanase (Glu).Bacterial cells were grown in NB medium

to early stationary phase (28°C), adjusted to an OD_{600} of 0.6, and 10 μL of the culture was inoculated onto each plate. After incubation at 28°C, enzyme activity was visualized by the formation of clear zones or colored halos, as described in the Methods section. The left column shows the full view of each plate, and the right column shows a magnified view of the inoculation site. Three independent experiments were performed, with consistent results observed across replicates.

Additional file 11:

Table S6 Identification of homologs of type II and Sec-SRP secretion system genes in *P.brasiliense* BS1113 and other *Pectobacterium* spp.

Additional file 12:

Table S7 Genetic elements of T6SS-encoding gene clusters in pathogenic *Pectobacterium* spp. were summarized and the presence of the key T6SS structure genes is indicated for the analysed genomes.

Additional file 13:

Table S8 Homolog of two-component system encoding genes in *P. brasiliense* BS1113 and other *Pectobacterium* spp.

Additional file 14:

Table S9 Homologs of Clustered regularly interspaced short palindromic repeats (CRISPR)-CRISPR-associated protein (Cas) in *P. brasiliense* BS1113 and other *Pectobacterium* spp.
