# Supplementary material for: Complete Genome Analysis of Pectobacterium brasiliense BS1113, a Causal Agent of Cigar Tobacco Soft Rot, with Phenotypic Characterization of Virulence and Copper Tolerance
Source: Genes (Basel). 2026 Jun 30;17(7):775. doi: 10.3390/genes17070775 (PMC13408941; doi:10.3390/genes17070775)
Supplement: Supplementary file 1 [file genes-17-00775-s001.zip › Additional file 6.pdf]

Table S4 Genome statistics

Attribute	Value	% of total
Genome size (bp)	4,916,962	100.00
DNA coding (bp)	4,261,943	86.67
DNA G + C (bp)	2,554,426	51.96
DNA scaffolds	1	100.00
Total genes	4,468	100.00
Protein coding genes (CDS)	4,369	97.78
RNA genes	99	2.22
Pseudo genes	Not reported	–
Genes in internal clusters	Not reported	–
Genes with function prediction	Not reported	–
Genes assigned to COGs	3,690	84.46
Genes with Pfam domains	Not reported	–
Genes with signal peptides	Not reported	–
Genes with transmembrane helices	Not reported	–
CRISPR repeats	6	–
