# Supplementary material for: Complete Genome Analysis of Pectobacterium brasiliense BS1113, a Causal Agent of Cigar Tobacco Soft Rot, with Phenotypic Characterization of Virulence and Copper Tolerance
Source: Genes (Basel). 2026 Jun 30;17(7):775. doi: 10.3390/genes17070775 (PMC13408941; doi:10.3390/genes17070775)
Supplement: Supplementary file 1 [file genes-17-00775-s001.zip › Additional file 5.pdf]

Table S3 Project information for *Pectobacterium brasiliense* strain BS1113

MIGS ID	Property	Term
MIGS 31	Finishing quality	Complete genome
MIGS-28	Libraries used	PacBio 20-kb SMRTbell library
MIGS 29	Sequencing platforms	PacBio RS II
MIGS 31.2	Fold coverage	861.0×
MIGS 30	Assemblers	SMRT Analysis v2.3.0
MIGS 32	Gene calling method	NCBI Prokaryotic Genome Annotation Pipeline (PGAP)
	Locus Tag	Not reported (or check GenBank entry)
	GenBank ID	CM128641.1
	GenBank Date of Release	Not reported (submitter-provided; contact NCBI)
	GOLD ID	Not reported
	BIOPROJECT	Not reported
MIGS 13	Source Material Identifier	BS1113
	Project relevance	Plant-bacteria interaction, soft rot pathogen of cigar tobacco
