# Supplementary material for: Complete Genome Analysis of Pectobacterium brasiliense BS1113, a Causal Agent of Cigar Tobacco Soft Rot, with Phenotypic Characterization of Virulence and Copper Tolerance
Source: Genes (Basel). 2026 Jun 30;17(7):775. doi: 10.3390/genes17070775 (PMC13408941; doi:10.3390/genes17070775)
Supplement: Supplementary file 1 [file genes-17-00775-s001.zip › Additional file 2.pdf]

Table S2 Classification and general features of Pectobacterium brasiliense strain BS1113 according to the MIGS recommendations.

MIGS ID	Property	Term	Evidence code[a]
	Classification		
	Domain	Bacteria	TAS
	Phylum	Proteobacteria	TAS
	Class	Gammaproteobacteria	TAS
	Order	Enterobacterales	TAS
	Family	*Pectobacteriaceae*	TAS
	Genus	*Pectobacterium*	TAS
	Species	*Pectobacterium brasiliense*	IDA
	Strain	BS1113	IDA
	Morphology		
	Gram stain	Negative	TAS
	Cell shape	Rod-shaped	TAS
	Motility	Motile	TAS
	Sporulation	Non-sporulating	TAS
	Physiology		
	Temperature range	Mesophilic (grows at 37°C)	IDA
	Optimum temperature	28°C	IDA
	pH range; Optimum	Not reported	NAS
	Carbon source	16 positive reactions on Biolog GEN III (detailed data in supplementary file)	IDA
MIGS-6	Habitat	Plants (Nicotiana tabacum cv. Yunxue 1), soil	IDA
MIGS-6.3	Salinity	Grows in 5% and 7% NaCl (w/v)	IDA
MIGS-22	Oxygen requirement	Facultatively anaerobic	TAS
MIGS-15	Biotic relationship	Free-living	TAS
MIGS-14	Pathogenicity	Pathogenic on Nicotiana tabacum cv. Yunxue 1 (cigar tobacco)	IDA
MIGS-4	Geographic location	Lujiang Town, Baoshan City, Yunnan Province, China	IDA
MIGS-5	Sample collection date	October 2022	IDA
MIGS-4.1	Latitude	24°48′–25°17′ N	IDA
MIGS-4.2	Longitude	98°45′–98°57′ E	IDA
MIGS-4.4	Altitude	Not reported	NAS

Evidence codes – IDA: Inferred from Direct Assay (e.g., Biolog phenotyping, physiological and biochemical tests, pathogenicity assays, strain isolation); TAS: Traceable Author Statement (based on published literature); NAS: Non-traceable Author Statement (not directly measured for this strain, based on general species characteristics or not reported).
