# Supplementary material for: Complete Genome Analysis of Pectobacterium brasiliense BS1113, a Causal Agent of Cigar Tobacco Soft Rot, with Phenotypic Characterization of Virulence and Copper Tolerance
Source: Genes (Basel). 2026 Jun 30;17(7):775. doi: 10.3390/genes17070775 (PMC13408941; doi:10.3390/genes17070775)
Supplement: Supplementary file 1 [file genes-17-00775-s001.zip › Additional file 1.pdf]

Table S1 Project information and sequencing statistics for *Pectobacterium brasiliense* BS1113

**Property**	**Term**
Finishing quality	Complete genome
Libraries used	PacBio 20-kb SMRT-bell library
Sequencing platforms	Illumina; PacBio RS II
Fold coverage	861.0-fold
Assemblers	PacBio Unicycler software v. 4.0.0
Gene calling method	NCBI Prokaryotic Genome Annotation Pipeline; GeneMarkS-2+
Locus Tag	ACU36R
Genbank ID	CM128641.1
GenBank Date of Release	September 30, 2025
BIOPROJECT	PRJNA1332297
Source Material Identifier	BS1113
Project relevance	Plant-bacteria interaction, tobacco pathogen
