# Supplementary figures and images for: Complete Genome Analysis of Pectobacterium brasiliense BS1113, a Causal Agent of Cigar Tobacco Soft Rot, with Phenotypic Characterization of Virulence and Copper Tolerance
Source: Genes (Basel). 2026 Jun 30;17(7):775. doi: 10.3390/genes17070775 (PMC13408941; doi:10.3390/genes17070775)

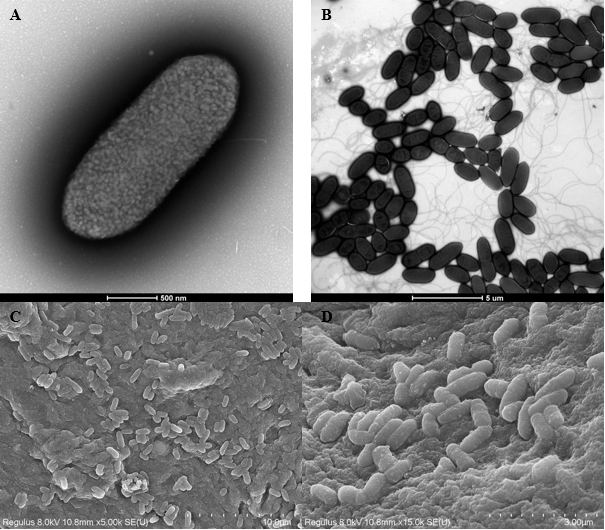

Supplement: Supplementary file 1 [file genes-17-00775-s001.zip › Additional file 3.png]

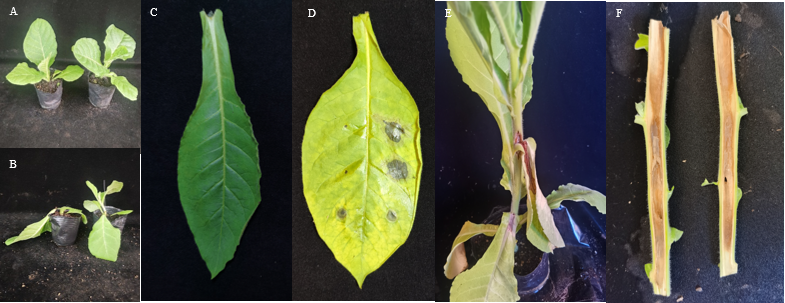

Supplement: Supplementary file 1 [file genes-17-00775-s001.zip › Additional file 4.png]

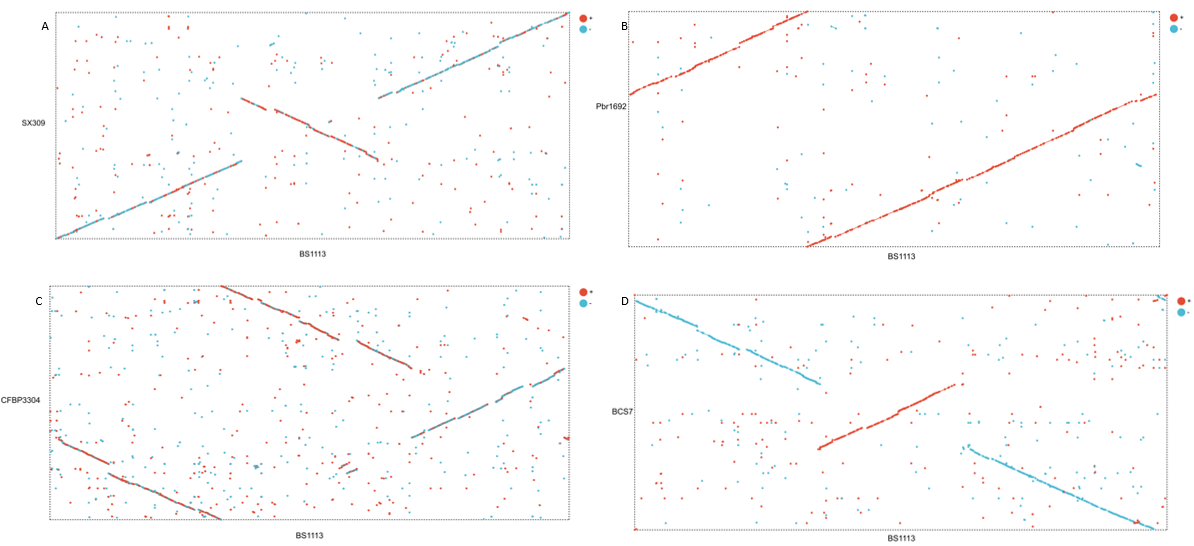

Supplement: Supplementary file 1 [file genes-17-00775-s001.zip › Additional file 10.png]

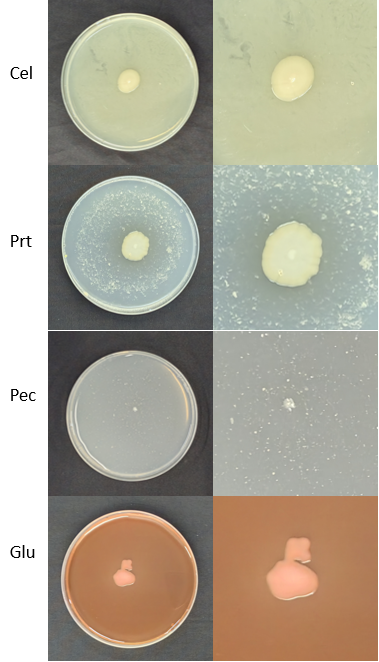

Supplement: Supplementary file 1 [file genes-17-00775-s001.zip › Additional file 11.png]
